# Optimizing window size and directional parameters of GLCM texture features for estimating rice AGB based on UAVs multispectral imagery

**DOI:** 10.3389/fpls.2023.1284235

**Published:** 2023-12-19

**Authors:** Jikai Liu, Yongji Zhu, Lijuan Song, Xiangxiang Su, Jun Li, Jing Zheng, Xueqing Zhu, Lantian Ren, Wenhui Wang, Xinwei Li

**Affiliations:** ^1^ College of Resource and Environment, Anhui Science and Technology University, Chuzhou, Anhui, China; ^2^ Anhui Province Crop Intelligent Planting and Processing Technology Engineering Research Center, Anhui Science and Technology University, Chuzhou, Anhui, China; ^3^ Institute of Agricultural Remote Sensing and Information, Heilongjiang Academy of Agricultural Sciences, Harbin, Heilongjiang, China; ^4^ School of Management, Heilongjiang University of Science and Technology, Harbin, Heilongjiang, China; ^5^ College of Life Science, Langfang Normal University, Langfang, Hebei, China; ^6^ College of Agriculture, Anhui Science and Technology University, Chuzhou, Anhui, China

**Keywords:** unmanned aerial vehicles (UAVs), aboveground biomass (AGB), multispectral imagery, texture features (TFs), grey level co-occurrence matrix (GLCM), rice

## Abstract

Aboveground biomass (AGB) is a crucial physiological parameter for monitoring crop growth, assessing nutrient status, and predicting yield. Texture features (TFs) derived from remote sensing images have been proven to be crucial for estimating crops AGB, which can effectively address the issue of low accuracy in AGB estimation solely based on spectral information. TFs exhibit sensitivity to the size of the moving window and directional parameters, resulting in a substantial impact on AGB estimation. However, few studies systematically assessed the effects of moving window and directional parameters for TFs extraction on rice AGB estimation. To this end, this study used Unmanned aerial vehicles (UAVs) to acquire multispectral imagery during crucial growth stages of rice and evaluated the performance of TFs derived with different grey level co-occurrence matrix (GLCM) parameters by random forest (RF) regression model. Meanwhile, we analyzed the importance of TFs under the optimal parameter settings. The results indicated that: (1) the appropriate window size for extracting TFs varies with the growth stages of rice plant, wherein a small-scale window demonstrates advantages during the early growth stages, while the opposite holds during the later growth stages; (2) TFs derived from 45° direction represent the optimal choice for estimating rice AGB. During the four crucial growth stages, this selection improved performance in AGB estimation with R^2^ = 0.76 to 0.83 and rRMSE = 13.62% to 21.33%. Furthermore, the estimation accuracy for the entire growth season is R^2^ =0.84 and rRMSE =21.07%. However, there is no consensus regarding the selection of the worst TFs computation direction; (3) Correlation (Cor), Mean, and Homogeneity (Hom) from the first principal component image reflecting internal information of rice plant and Contrast (Con), Dissimilarity (Dis), and Second Moment (SM) from the second principal component image expressing edge texture are more important to estimate rice AGB among the whole growth stages; and (4) Considering the optimal parameters, the accuracy of texture-based AGB estimation slightly outperforms the estimation accuracy based on spectral reflectance alone. In summary, the present study can help researchers confident use of GLCM-based TFs to enhance the estimation accuracy of physiological and biochemical parameters of crops.

## 1 Introduction

Rice (Oryza sativa) serves as a vital staple crop, nourishing almost half of the global population (Seck et al., 2012). In China, as the largest rice producer, consumer, and importer globally, rice constitutes a staple food for approximately 65% of the population (Peng et al., 2009). Accurate and timely prediction of rice yield holds significance in stabilizing rice prices, enhancing global food security, and enabling decision-makers to formulate timely strategies for rice import and export (Spiertz and Ewert, 2009; Cao et al., 2021). Aboveground biomass (AGB), the total dry matter per unit area of land during a specific period, plays a crucial role in understanding crop growth and development. AGB is an essential agronomic parameter to describe crop growth and nutritional status, making it instrumental in predicting crop yield (Li et al., 2020; Li et al., 2022). Thus, rapid and non-destructive assessment of the spatiotemporal dynamics of crop AGB is necessary for formulation and implementation of decision management and yield prediction in the field.

Although the field destructive sampling method is highly accurate, the inherent limitations in terms of time consumption and inefficiency render them inadequate for the timely, rapid, and large-scale requirements of modern agricultural monitoring (Fu et al., 2021). In contrast, the rapid development of remote sensing has presented new opportunities for non-destructive monitoring of AGB information in crop fields (Lu et al., 2019). Multispectral or hyperspectral images acquired from satellites or near-ground platforms offer a rapid means of monitoring AGB in dynamic spatiotemporal contexts. These platforms have been widely used to monitor various crop information, including wheat (Bao et al., 2009; Fu et al., 2014; Teng et al., 2015), rice (Gnyp et al., 2014; Cheng et al., 2017; Alebele et al., 2020), corn (Liao et al., 2019; Naidoo et al., 2021), and rapeseed (Mercier et al., 2020a; Mercier et al., 2020b). However, the continuous and prolonged monitoring of AGB has limitations due to the unavailability of satellite data during critical growth stages caused by weather variability and longer data return cycles (Gan et al., 2023). Additionally, the relatively low spatial resolution of satellite data hampers the acquisition of precise details about the crop canopy (Jin et al., 2020). On the contrary, near-ground platforms, which emphasize plant-to-sensor and sensor-to-plant models, provide certain advantages, such as continuous monitoring and payload availability. However, the limitations in terms of monitoring throughput and data acquisition scale impede their ability to capture crop AGB information at large scales (Lakhiar et al., 2018; Qiu et al., 2018; Jangra et al., 2021). The Unmanned aerial vehicles (UAVs) platform is the latest hot topic for research on crop phenotypes. It can be equipped with multi-source sensors to acquire multi-source remote sensing information, making it an effective supplement to both satellite and near-ground remote sensing platforms. UAVs provide high-precision data support and efficient monitoring capabilities, facilitating non-destructive monitoring of crop AGB (Maes and Steppe, 2019; Mukherjee et al., 2019).

Extensive research has been conducted utilizing spectral information from UAVs imagery to monitor crop AGB, and it has achieved acceptable monitoring accuracy (Han et al., 2019; Maimaitijiang et al., 2019; Wang F. et al., 2022; Wang et al., 2023). However, spectral information is susceptible to interference from water-soil (and weed) background noise during the early stages, while the dense canopy coverage at the later stages can result in spectral saturation (Li et al., 2022; Wang F. et al., 2022; Wang Q. et al., 2022; Wang W. et al., 2022; Zhu et al., 2023). These factors impose constraints on further advancements in monitoring accuracy. To address the issue of low accuracy in monitoring AGB based on spectral information, an increasing number of researchers are inclined toward utilizing texture features (TFs) to improve the precision of crop AGB monitoring (Liu Y. et al., 2019; Yue et al., 2019; Wang F. et al., 2022; Xu T. et al., 2022). TFs describe the frequency of variations in attribute values among adjacent pixel pairs within a specific window (Liu Y. et al., 2019; Yue et al., 2019; Zhang et al., 2021). They can provide complementary information regarding the spatial arrangement and patterns of crop canopy, which helps overcome the limitations of spectral information (Liu Y. et al., 2019; Zhang et al., 2021). The color differences in crop leaves caused by factors such as crop varieties and soil nutrients can be captured by TFs (Zhang et al., 2021; Wang F. et al., 2022). Furthermore, due to crops undergoing growth and development, changes in canopy structure also generate texture variations. For instance, the emergence of rice panicles from leaf sheaths, the flowering of spikelets, the gradual drooping of mature panicles, and variations in the proportion between rice plant and background or shadows all contribute to subtle changes in canopy morphology. These subtle variations in canopy structure inevitably result in alterations in TFs (Yue et al., 2019; Xu L. et al., 2022). Incorporating TFs into the analysis provides a robust means of capturing subtle variations and improving the accuracy of crop AGB monitoring in agricultural remote sensing studies. Many studies utilize the gray-level co-occurrence matrix (GLCM) to extract TFs from UAVs images (Lu, 2005; Dube and Mutanga, 2015; Liu et al., 2018; Liang et al., 2022; Xu L. et al., 2022). By incorporating GLCM-based TFs into AGB monitoring, researchers have improved the accuracy of forest and crop biomass estimation. For example, Kelsey and Neff (Kelsey and Neff, 2014) discovered that forest AGB estimation models incorporating TFs exhibited greater accuracy compared to models relying solely on spectral information. Zheng et al. (2019) demonstrated that the incorporation of TFs can significantly enhance the monitoring accuracy of rice AGB, particularly during the mid to late stages of the rice growth season.

Existing research has demonstrated that window size and direction texture parameters are highly sensitive to texture metrics when extracting TFs using the GLCM (Zheng et al., 2020; Liang et al., 2022; Zhou M. et al., 2023). Throughout the entire growth season, the spatiotemporal fluctuations in the rice canopy coverage determine the requisite selection of appropriate window size for quantifying texture disparities. Furthermore, as rice is a row-cropped crop with an apparent spatial direction, the directional selection may impinge upon the monitoring performance of TFs in assessing AGB. However, previous studies have primarily relied on default texture parameters setting (such as a 3x3 window size and diagonal direction at 45°) (Zheng et al., 2018; Li et al., 2019; Zheng et al., 2019; Xu L. et al., 2022; Zhang D. et al., 2022; Zhang Y. et al., 2022;) or a directionless approach (by averaging multiple directional TFs to eliminate the directional effect) (Wang F. et al., 2021; Liu et al., 2022a; Xu T. et al., 2022) when extracting GLCM-based TFs. A quantitative analysis of the impact of TFs derived from different window sizes and direction parameters for crop AGB estimation has been omitted from these studies.

Fortunately, a limited amount of research has recently emerged that focuses on the impact of texture parameters on the accuracy of AGB monitoring. For example, Fu et al. (2021) and Yue et al. (2019) had demonstrated that the impact of window size and directional parameters on winter wheat AGB estimation was relatively minor. Zheng et al. (2020) discovered a conspicuous directional effect in rice texture information, with the direction parallel to the planting rows (i.e., the 0° direction of GLCM) being the optimal direction for monitoring rice nitrogen content. For monitoring potato AGB, contrasting conclusions had been drawn by Liu et al. (2022a) and Luo et al. (2022). Liu et al. considered directional parameters could be negligible for estimating AGB, whereas Luo et al. considered the 45° direction optimal for texture extraction. Although some studies have confirmed the sensitivity of crop AGB to TFs related to window size and directional parameters, further research in this area is still lacking in comprehensiveness. Given the wide use of GLCM-based TFs in crop growth monitoring, there is an urgent need to develop a deeper understanding of how texture parameters impact rice AGB monitoring. To address this, our study utilizes Random Forest (RF) regression model to investigate the influence of GLCM texture parameters on the monitoring accuracy of rice AGB during critical growth stages. The specific objectives of our study are as follows: 1) to evaluate how texture window size affects the accuracy of rice AGB monitoring and determine the suitable window size to estimate AGB; 2) to assess the impact of the directional parameter on the accuracy of rice AGB monitoring and identify the optimal direction for texture extraction; and 3) to explore the significance of TFs for rice AGB estimation and interaction mechanistic.

## 2 Materials and methods

### 2.1 Experimental design

**Figure 1:**
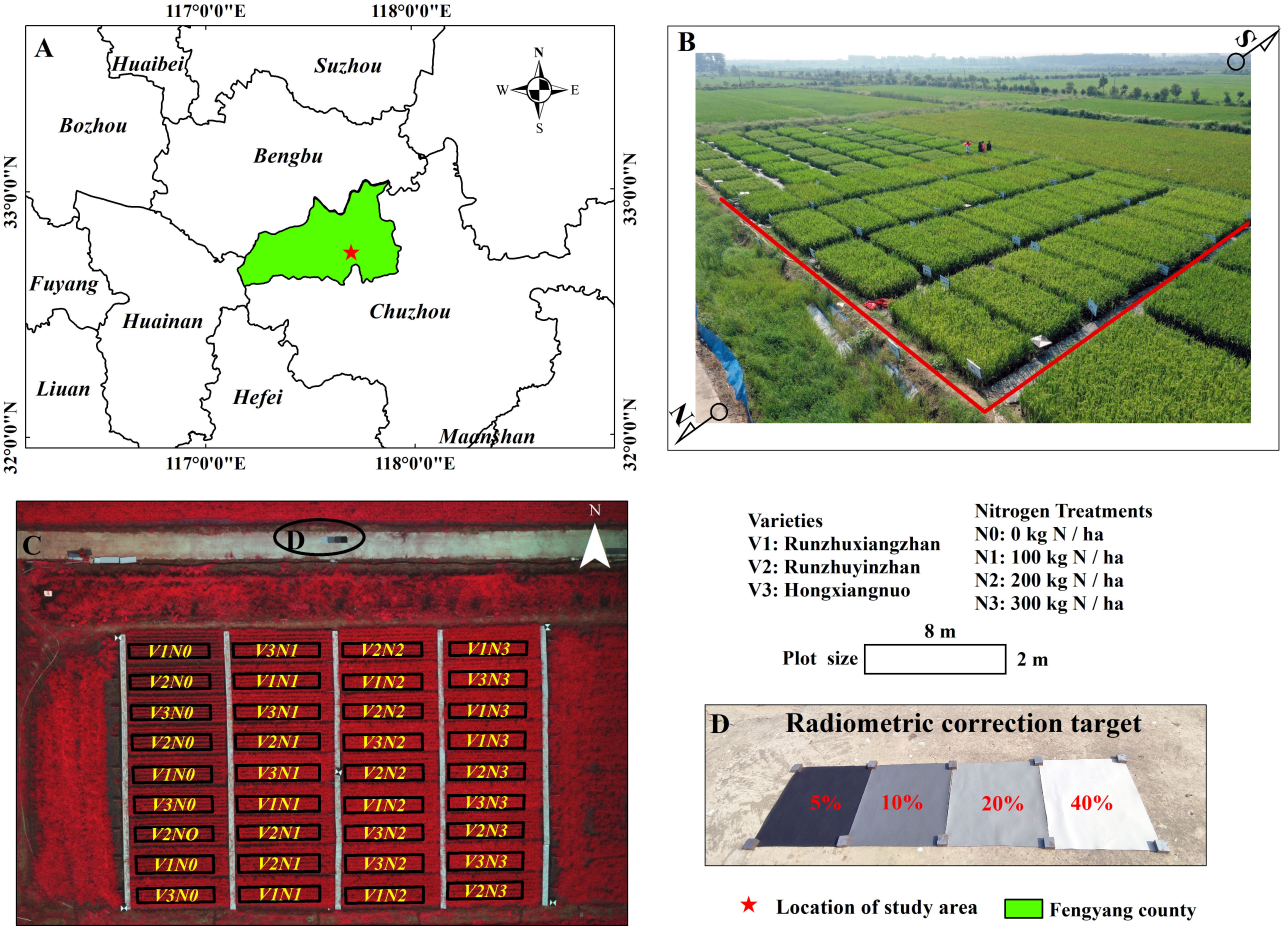
Location of study area **(A)**, field experimental design **(B, C)**, and radiometric correction target **(D)**.

A field experiment was conducted at the modern agricultural research institute, Anhui Science and Technology University located in Xiaogang Village, Fengyang County, Anhui Province, China (117°42’ E, 32°16’ N) (**Figure 1A**), where the climate belongs to a transitional pattern from the northern subtropical to the temperate zone. The average annual rainfall in 2020 was 1179.2 mm, and the average annual temperature was 15.5°C (Station No. 58222, Fengyang County Meteorological Station). The experiment design involved four N fertilizer treatments (N0: 0 kg/ha; N1: 100 kg/ha; N2: 200 kg/ha, and N3: 300 kg/ha) and three rice varieties (V1: RunzhuXiangzhan, V2: RunzhuYinzhao, and V3: Hongxiangnuo). A randomized complete block design was adopted for the experiment field, with N fertilizer treatments as the main plot and rice varieties as the split plot. There were three replications, leading to a total of 36 subplots, measuring 2 m × 8 m each, and the double-layer impermeable plastic film was used to isolate different nitrogen fertilizer treatments plot (**Figures 1B, C**). The experiment started in May 2020 with land preparation and delineation of plots, followed by basal fertilizer application and irrigation. Seeding took place on May 24th, and transplanting was conducted on June 23rd. The rice seedlings were manually inserted at a spacing of 30 cm between rows and 15 cm between plants, with one seedling per hill (including 1-2 tillers). In mid-July, an additional application of topdressing fertilizer was administered during the tillering stage. Subsequently, in early August, the plants entered the heading and panicles initiation stage, and on August 23rd, another additional fertilization was applied. The early grain-filling stage began in early September, and harvest was conducted on October 2nd. Phosphorus fertilizer (CaP_2_H_4_O_8_, with available P_2_O_5_ content of 12% and a pure phosphorus equivalent of 90 kg/ha) and potassium fertilizer (KCl, with available K_2_O content of 60% and a pure potassium equivalent of 135 kg/ha) were applied as basal fertilizers. Nitrogen fertilizer (urea/CH_4_N_2_O, with an available nitrogen content of 46%) was applied in three stages, with a ratio of 4:3:3 for basal, tillering, and panicle fertilizer. Field management followed local high-yield cultivation techniques, including the application of herbicides and pesticides as general practices in this area.

### 2.2 Main workflow

**Figure 2:**
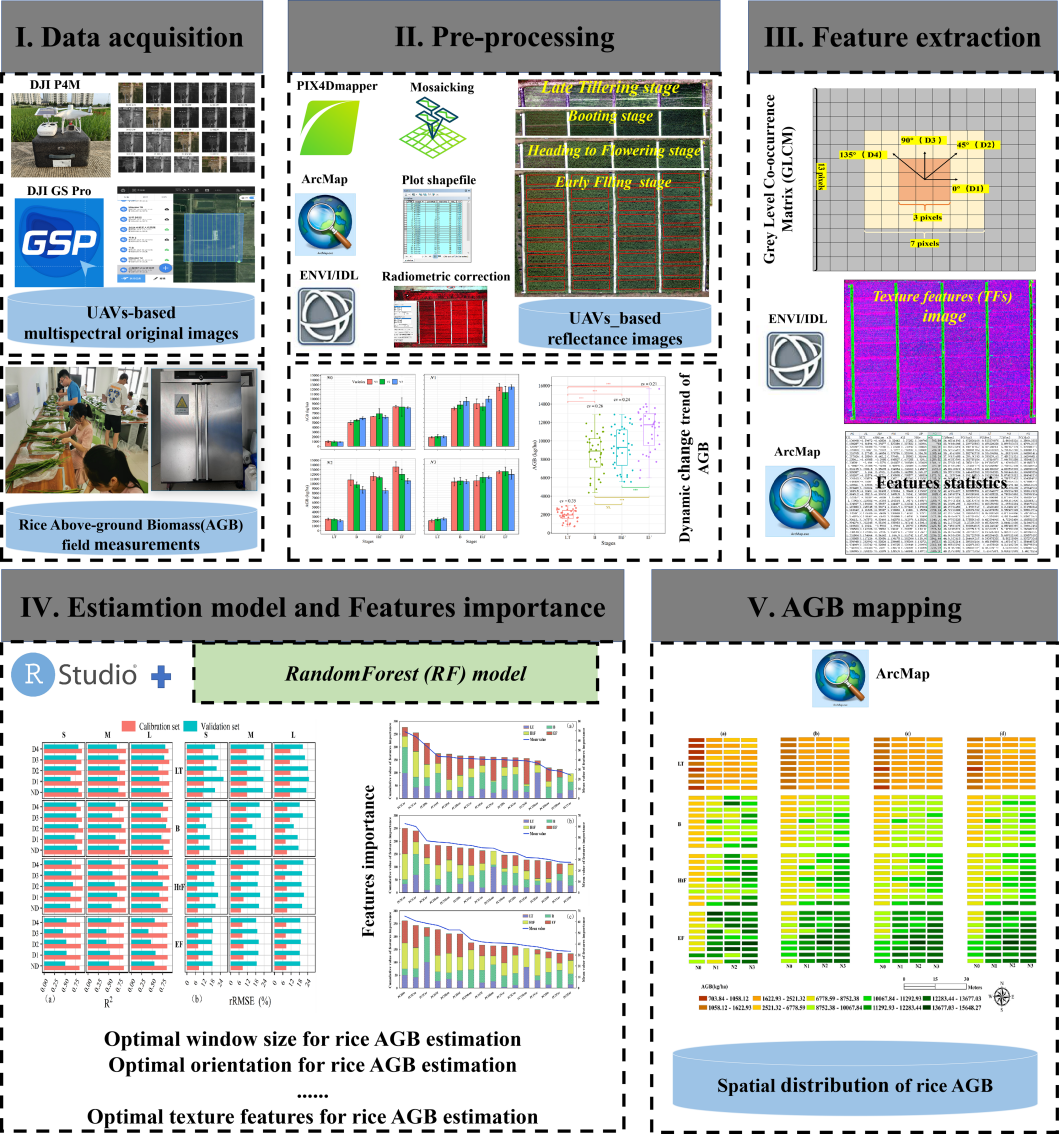
Flowchart of this study.

**Figure 2** shows the flowchart describing the procedures for estimating rice AGB from UAVs-based multispectral imagery. This process consisted of five main aspects: (1) acquiring UAVs remote sensing images and ground-truth AGB measurements; (2) preprocessing the acquired data in step I; (3) extracting TFs using various window sizes and directional parameters combination; (4) Random Forest (RF) regression model was built based on TFs calculated from step III for rice AGB estimation, and analyzed the significance of the TFs; (5) Estimating results were used to map AGB during the whole growth season.

### 2.3 Data acquisition and preprocessing

#### 2.3.1 UAVs data acquisition and preprocessing

The DJI Phantom 4 Multispectral RTK (P4M) UAVs (DJI, Shenzhen, Guangdong, China) was used to acquire multispectral images at four growth stages, including the late tillering stage (LT: 25/07/2020), booting stage (B: 23/08/2020), heading to flowering stage (HtF: 31/08/2020), and early filling stage (EF: 09/09/2020) (**Table 1**). The P4M equipped with five monochrome sensors are used for multispectral images (blue (450nm±16nm, B), green (560nm±16nm,G), red (650nm±16nm, R), red edge (730nm±16nm, RE), and near-infrared (840nm±26nm, NIR) image). Among them, the RE and the NIR are the important selection of crop parameter inversion (Cui and Kerekes, 2018a; Liu J. et al., 2022).The multispectral sensors record each monochrome for 2.12-megapixel with a 40-mm focal length and 62.7-degree field of view. The multispectral image was geotagged automatically by its built-in multi-frequency high-precision RTK GNSS positioning system which provides about 8 mm and 15 mm accuracy in the vertical and horizontal directions, respectively. **Supplementary Table 1** describes the technical specifications of the P4M.

**Table 1:** The flight details during the whole growth season.

Dates	Growth stages	Height(m)	Spatial resolution (cm*cm)
**07/25/20**	Late Tillering (LT)	30	1.70
**08/23/20**	Booting (B)		
**08/31/20**	Heading to Flowering (HtF)		
**09/09/20**	Early Filling (EF)		

Flight campaigns were planned to utilize DJI GS Pro software, which allows autonomous path points to be defined by the user in all flight campaigns. All campaigns were conducted on cloud-free days from 10:00–12:00 A.M. (local time) and were planned at a flight altitude of 30 m and flight speed of 3 m/s with a forward overlap of 90% and a side overlap of 85%. The flight campaigns were kept consistent throughout the growth season to ensure consistency in data collection.

PIX4Dmapper software (Pix4D SA, Lausanne, Switzerland, https://www.pix4d.com/) was used to generate orthophoto images from four critical growth stages (Wang F. et al., 2022). Images were aligned based on key points, then mosaicked and generated dense point clouds. To build high-density point clouds, “Half image size” for the image scale option and “Optimal” for the point density option were selected. Subsequently, a textured mesh was generated based on the constructed point cloud, resulting in the production of the Digital Surface Model (DSM) and Digital Orthophoto Image (DOM).

Due to the absence of spectral response function (SRF) of camera, it is not possible to quantify the influence of SRF (Cui and Kerekes, 2018b). Hence, we employ empirical linear model (ELM) for radiometric correction purposes (Di Gennaro et al., 2022; Liu J. et al., 2022). Four standard diffuse panels (**Figure 1D**) were placed on one side of the study area within the UAVs field of view (**Figure 1C**). The reflectance of each diffuse panel was measured using the ASD FieldSpec HandHeld2 portable spectrometer (Analytical Spectral Devices, Boulder, Colorado, USA). The digital number (DN) values of the UAVs images were converted into reflectance values using ELM to eliminate radiometric distortions caused by variations in lighting conditions during different stages. The ELM is conducted through the following Equation (1).


(1) $$R_{i}=DN_{i}\times Gain_{i}+Offset_{i}\begin{array}{c} \left(i=1,2,\ldots ,5\right) \end{array}$$

where, $R_{i}$ and $DN_{i}$ respectively represent the reflectance values and original DN values corresponding to the i th band in the multispectral image. $Gain_{i}$ and $Offset_{i}$ respectively represent the conversion coefficient corresponding to the i th band, which are calculated using the ordinary least squares (OLS) method.

In order to conduct analysis and modeling at the plot level, the unique shapefiles, which removed inward a row of rice plant, were created manually using ArcGIS(10.2 version, Environmental Systems Research Institute, Inc., Redlands, CA, USA) (**Figure 1C**). This approach was implemented to account for the vigorous growth of rice plant at the margin, which can be influenced by incomplete spatial constraints or competition with contiguous rows. Failing to mitigate such boundary effects could lead to an overestimation of spectral reflectance within the plot.

#### 2.3.2 AGB measurement

Following the collection of UAVs data, we randomly sampled 3 hill plants from each plot by cutting the stems approximately 2 cm above the soil surface and the stems, leaves, and panicles would be separated for AGB measurements. The separated samples were then oven-dried at 105°C for 30 min and then at 75°C until weights stabilized. Dry samples were weighed and summed to obtain the aboveground dry biomass. Finally, 144 AGB measurements were collected in total (four growth stages) and converted into a unified value of kg/ha based on the planting row and plant spacing.

### 2.4 Feature extraction

Texture analysis methods can be categorized into four types: statistical method, structural method, model-based method, and transformation-based method (Haralick et al., 1973; Hall-Beyer, 2007; Hall-Beyer, 2017). The most commonly used method is based on the Gray-Level Co-occurrence Matrix (GLCM), which was first introduced by Haralick in 1973 to reveal the variation properties of the spatial distribution of grayscale values in an image at a certain distance (d) and specific angle (θ) (Pacifici et al., 2009). GLCM ensures non-deformation, rotation-invariant multi-scale features, and low computational complexity. In this study, eight TFs were selected: Mean (Mean), Variance (Var), Homogeneity (Hom), Contrast (Con), Dissimilarity (Dis), Entropy (Ent), Second Moment (SM), and Correlation (Cor) (**Table 2**).

**Table 2:** Texture features of multispectral imagery (Hall-Beyer, 2007; Pacifici et al., 2009; Hall-Beyer, 2017).

Texture features	Calculation equations	Features description
**Mean (Mean)**	$Mean=\sum_{i=1}^{N_{g}}\sum_{j=1}^{N_{g}}i\times P(i,j)$	The mean value in the GLCM window.
**Variance(Var)**	$Var=\sum_{i=1}^{N_{g}}\sum_{j=1}^{N_{g}}{\left(i-u\right)}^{2}\times P(i,j)$	The variance in the GLCM window.
**Homogeneity(Hom)**	$Hom=\sum_{i=1}^{N_{g}}\sum_{j=1}^{N_{g}}\frac{1}{1+{\left(i-j\right)}^{2}}\times P(i,j)$	The homogeneity of grey level in the GLCM window.
**Contrast(Con)**	$Con=\sum_{i=1}^{N_{g}}\sum_{j=1}^{N_{g}}{\left(i-j\right)}^{2}\times P(i,j)$	The clarity of texture in the GLCM window, as opposed to HOM.
**Dissimilarity(Dis)**	$Dis=\sum_{i=1}^{N_{g}}\sum_{j=1}^{N_{g}}\left|i-j|\times P(i,j\right)$	The similarity of the pixels in the GLCM window, similar to CON.
**Entropy (Ent)**	$Ent=-\sum_{i=1}^{N_{g}}\sum_{j=1}^{N_{g}}P(i,j)\times \log\left[P(i,j)\right]$	The diversity of the pixels in the GLCM window, proportional to the complexity of the image texture.
**Second Moment (SM)**	$SM=\sum_{i=1}^{N_{g}}\sum_{j=1}^{N_{g}}{\left[P(i,j)\right]}^{2}$	The uniformity of greyscale in the GLCM window.
**Correlation(Cor)**	$Cor=\frac{\sum_{i=1}^{N_{g}}\sum_{j=1}^{N_{g}}(i,j)\times P(i,j)-u_{i}u_{j}}{\sigma_{i}\sigma_{j}}$	The linear dependency of greyscale on those of neighboring pixels in the GLCM window.

P(i,j) represents the probability of each pixel pair (i,j) value and i, j are the gray tones in the windows, which are also the coordinates of the co-occurrence matrix space; Ng represents the number of distinct grey levels in the quantized image, which has a gray value range of the original image; μ and σ represents the mean and standard deviation of P(i,j), respectively.

**Figure 3:**
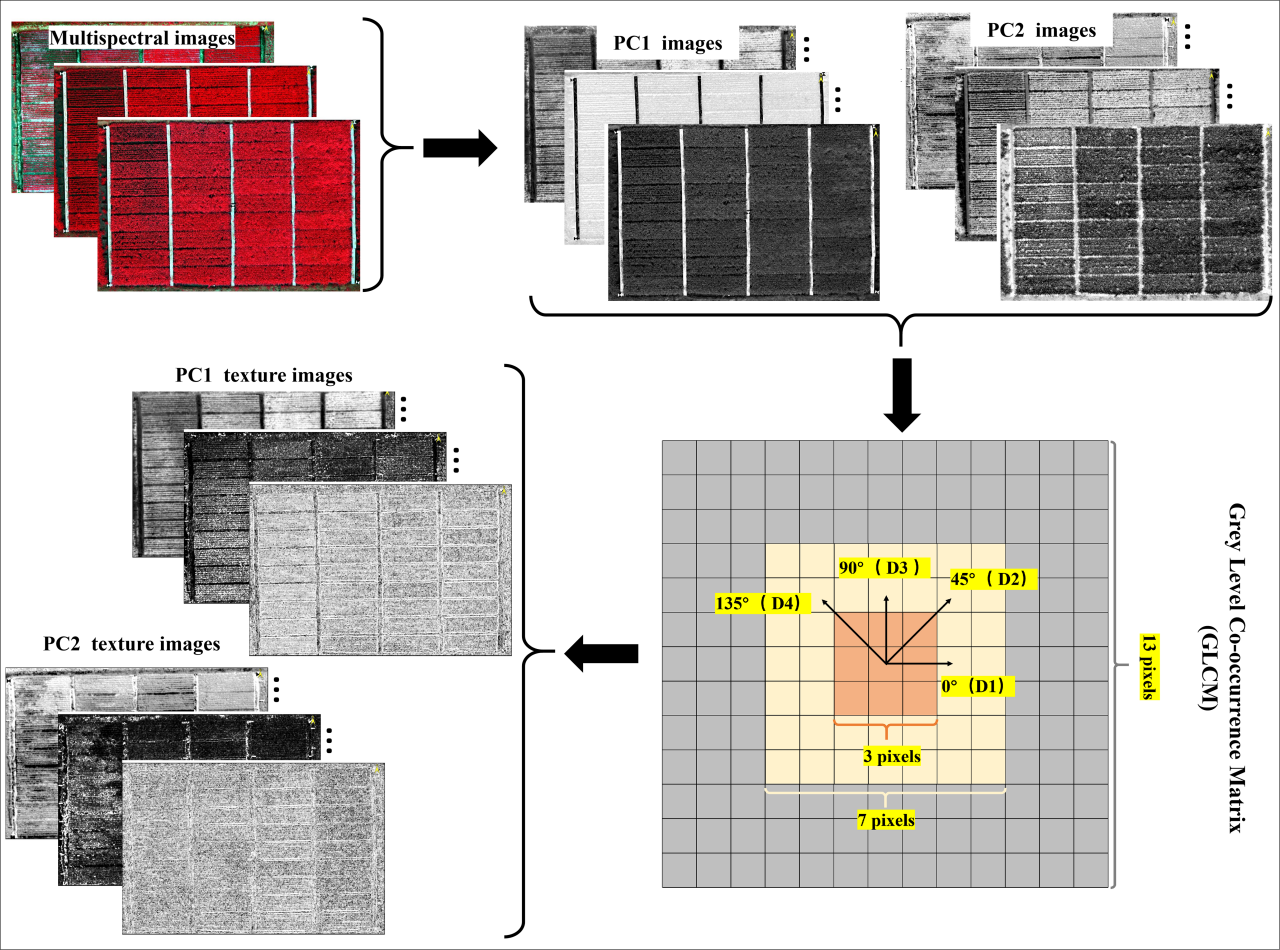
Details of GLCM-based texture features calculation.

The GLCM requires users to define the window size, direction, and displacement of the moving window. In this study, the displacement was set to 1 as it is the most commonly used setting (Liu Y. et al., 2019; Zheng et al., 2019; Wang Q. et al., 2022; Xu L. et al., 2022). To determine and select the most optimal texture parameters, TFs for all multispectral images were calculated using three window sizes (3 × 3, 7 × 7, and 13 × 13 pixels, denoted as S, M, and L, respectively) and four directions (0°, 45°, 90°, and 135°, denoted as D1, D2, D3, and D4, respectively). The average TFs values of these four directions were then obtained to achieve rotation invariance, referred to as the non-directional (ND) texture metrics. **Figure 3** depicts more details on the selection of window sizes and directions for TFs calculation.

All TFs were calculated from the component images after principal component analysis (PCA). On one hand, this choice was made to reduce redundancy among the multispectral data; on the other hand, to minimize the computational workload during GLCM construction (Liu C. et al., 2019). In this study, the principal components with a cumulative contribution rate exceeding 90% were selected (**Supplementary Figure 1**), specifically the first two principal components from the PCA analysis of the multispectral imagery. After calculation, a total of 240 [2 (principal components of PCA) × 8 (texture features) × 3 (window sizes) × 5 (directions) = 240] TFs were generated for each growth stage.

### 2.5 AGB estimation model

#### 2.5.1 Random Forest model

Random Forest (RF) is an ensemble learning algorithm proposed by Breiman and is based on multiple decision trees and Bagging technology (Breiman, 2001). In the model, decision trees are built in parallel, with each tree trained on a different subset of data. Thus, each decision tree is unique, reducing the model’s variance and lowering prediction errors (Yu et al., 2016). For regression models, the main advantages of RF are as follows: 1) lack of sensitivity to collinearity among multiple variables; 2) presence of few parameters that require tuning, with only one hyperparameter in this study; 3) effective reduction of the risk of overfitting; 4) automatic calculation of variable importance scores to assess the contribution of individual predictors to the model (Liu Y. et al., 2019; Burdett and Wellen, 2022; Borrmann et al., 2023).

The RF model comprises two crucial hyperparameters: the number of decision trees (ntree) and the number of input variable features at each node (mtry). When adjusting ntree to a sufficiently large value, it primarily impacts the modeling time rather than the modeling accuracy (Wang et al., 2016; Zhang et al., 2021). Therefore, following its application in other studies, we set ntree to 1,000 (Li et al., 2019; Zhu et al., 2023). On the other hand, the value of mtry significantly affects the modeling accuracy of RF. Thus, it should be adjusted based on the number of input variable sets to optimize the RF model. The determination of the parameter mtry involves a grid search for parameter optimization.

#### 2.5.2 Accuracy assessment

Cross-validation (CV) was employed based on 70% of samples to determine the AGB monitoring model with the highest determination coefficient (R^2^) and lowest root mean squared error (RMSE) for improving the model’s stability. The RF model was then evaluated on the remaining 30% of samples(**Supplementary Table 2**). This approach effectively enhances the applicability of the RF algorithm on small datasets.

Richter et al. (2012) recommends a set of statistical test metrics that can comprehensively quantify the performance of models through literature review and experimental calculation. In this study, we adopt three recommended metrics: R^2^, RMSE, and relative root mean squared error (rRMSE). Their calculation formulas are presented as Equations (2–4)).


(2) $$R^{2}=\frac{\sum_{i=1}^{n}\left(AGB_{obs}^{i}-\bar{AGB_{obs}})\times (AGB_{est}^{i}-\bar{AGB_{obs}}\right)}{\sqrt{\sum_{i=1}^{n}{\left(AGB_{obs}^{i}-\bar{AGB_{obs}}\right)}^{2}\times \sum_{i=1}^{n}{\left(AGB_{est}^{i}-\bar{AGB_{obs}}\right)}^{2}}}$$

(3) $$RMSE=\sqrt{\frac{1}{n}\sum_{i=1}^{n}{\left(AGB_{est}^{i}-AGB_{obs}^{i}\right)}^{2}}$$

(4) $$rRMSE=100\times \frac{RMSE}{\bar{AGB_{obs}}}$$

where n represents the number of samples in RF model; $AGB_{obs}^{i}$ and $AGB_{est}^{i}$ represent the truth ground AGB measurement and the estimated AGB value of i sample, respectively; and $\bar{AGB_{obs}}$ and $\bar{AGB_{est}}$ represent the average truth ground AGB measurement and the average estimated AGB value of all samples, respectively.

The normalized increase in mean square error (%IncMSE) ranging from 0 to 100 is used to assess the importance of variables in the RF model. %IncMSE is calculated by permuting out-of-bag (OOB) data, where higher percentages indicate greater importance of variables. For a detailed description of %IncMSE, please refer to the literature (Breiman, 2001). All data analyses were conducted using R programming language (https://www.r-project.org) in RStudio software (Version 4.2).

## 3 Results

### 3.1 Effects of growth stages and nitrogen levels on rice AGB

**Figure 4:**
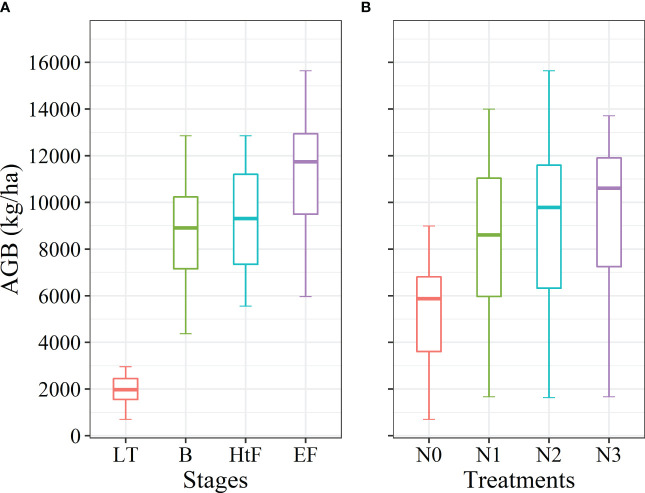
Rice AGB variation with **(A)** growth stages and **(B)** N treatments over the experimental plots. LT, Late Tillering stage; B, Booting stage; HtF, Heading to Flowering stage; EF, Early Filling stage.

**Figure 4** shows the changes in rice AGB of all rice plots under different growth stages and treatments. AGB increased rapidly with the development of growth stages, ranging from 1921.42 kg/ha to 11179.76 kg/ha, with a standard deviation of 4,009.28 kg/ha. The coefficient of variation (CV) exhibited a gradual decline during the crop growth stages, diminishing from 0.35 at the LT stage to 0.21 at the EF stage (**Figure 4A** and **Supplementary Table 3**). With the increase of N fertilization, the AGB exhibited the same change pattern as the growth stage, yet CV values were higher between different nitrogen levels (CV:0.45 ~ 0.55) (**Figure 4B** and **Supplementary Table 4**).

### 3.2 Effects of the window size parameter on the accuracy of rice AGB estimation

To determine the appropriate window size for extracting TFs from multispectral PCA images (PC1 and PC2 images), we calculated TFs using three different window sizes. The RF estimation model integrated 240 extracted TFs to evaluate the window size that yielded the most optimal model performance (**Table 3**). Notably, the appropriate window size exhibited dynamic variations across different growth stages. Small window size demonstrated superior performance during the vegetative growth period, while large window size performed better during the reproductive growth period. Interestingly, the monitoring accuracy of AGB was the lowest when TFs derived from a medium-size window were used during the four crucial growth periods. At the LT stage, the highest precision for AGB monitoring was achieved from a small window size, with R^2^=0.82, RMSE=378.64 kg/ha, and rRMSE=20.3%. Similarly, the best accuracy of R^2^=0.82, RMSE=1173.01 kg/ha, and rRMSE=13.62% was also achieved from a small window size at the B stage. At the HtF and EF stages, the highest estimation accuracy was achieved both from large window size that R^2^=0.75, RMSE=1658 kg/ha and rRMSE=19.42% and R^2^=0.58, RMSE=1848.66 kg/ha and rRMSE=18.28%, respectively.

**Table 3:** Estimation accuracy of rice AGB model during the critical growth stages based on texture features.

Windows	Directions	Late Tillering stage			Booting stage			Heading to Flowering stage			Early Filling stage		
		RMSE (kg/ha)	R^2^	rRMSE (%)	RMSE (kg/ha)	R^2^	rRMSE (%)	RMSE (kg/ha)	R^2^	rRMSE (%)	RMSE (kg/ha)	R^2^	rRMSE (%)
**Small**	ND	376.35	0.82	20.17	1401.00	0.79	16.27	1761.93	0.62	20.64	1869.57	0.50	18.49
	D1	491.46	0.63	26.35	1410.43	0.73	16.38	1918.14	0.63	22.47	1872.06	0.54	18.51
	D2	399.85	0.76	21.43	1173.01	0.82	13.62	1664.21	0.69	19.50	1813.24	0.53	17.93
	D3	421.89	0.77	22.62	1552.01	0.77	18.02	1686.04	0.65	19.75	1908.85	0.45	18.88
	D4	378.64	0.82	20.3	1511.91	0.77	17.56	1779.76	0.66	20.85	1826.19	0.54	18.06
**Middle**	ND	431.71	0.73	23.14	1577.41	0.74	18.32	1744.06	0.57	20.43	1897.33	0.51	18.76
	D1	436.52	0.7	23.40	1574.8	0.66	18.29	1808.40	0.70	21.19	2019.91	0.53	19.98
	D2	397.88	0.76	21.33	1335.28	0.80	15.51	1658.65	0.64	19.43	2038.48	0.44	20.16
	D3	442.94	0.73	23.74	1866.81	0.58	21.68	1720.56	0.59	20.16	1928.3	0.44	19.07
	D4	443.72	0.69	23.79	1798.19	0.66	20.88	1793.00	0.57	21.00	1848.5	0.56	18.28
**Large**	ND	426.54	0.71	22.87	1490.65	0.77	17.31	1691.00	0.71	19.81	1870.34	0.55	18.5
	D1	461.52	0.67	24.74	1425.64	0.72	16.56	1802.98	0.67	21.12	1848.66	0.58	18.28
	D2	453.15	0.65	24.29	1198.83	0.83	13.92	1658.00	0.75	19.42	1919.27	0.47	18.98
	D3	410.27	0.79	21.99	1766.66	0.71	20.52	1698.83	0.64	19.90	1949.16	0.41	19.28
	D4	381.10	0.81	20.43	1572.64	0.76	18.26	1774.50	0.64	20.79	1879.00	0.56	18.58

### 3.3 Effects of the direction parameter on the accuracy of rice AGB estimation

TFs possess inherent directional properties, and the influence of directional parameters on rice AGB estimation was complex (**Table 3**). Taking into account the 4 growth stages, 3 window sizes, and 5 directions, there was a three-fourth probability (among the 12 optimal models, 8 models achieved the highest accuracy) that AGB estimation models exhibited greater accuracy in the D2 direction (45°), especially during the intermediate periods of rice growth (from B stage to HtF stage in this study). Taking the example of large window size, at the B stage, the highest accuracy for AGB estimation was achieved in the D2 direction, with R^2^ = 0.83, RMSE = 1,198.83 kg/ha, and rRMSE = 13.92%. Similarly, at the HtF stage, the highest accuracy for AGB estimation was also observed in the D2 direction, with R^2^ = 0.75, RMSE = 1,658.00 kg/ha, and rRMSE = 19.42%. Acceptable estimation accuracy was achieved for rice AGB in the D4 direction (135°, orthogonal to the D2 direction) as well. In contrast, the poorest performing directions did not exhibit a consistent pattern, as it occurred with a probability of 5 out of 12 optimal models in both D1 (0°) and D3 (90°) directions. Taking the large window size as an example, the TFs from D1 direction exhibited the lowest monitoring accuracy for AGB during the LT and HtF stages, with R^2^ =0.67 and 0.67, and rRMSE = 24.74% and 21.12%, respectively. Moreover, the TFs from the D3 direction demonstrated the lowest monitoring accuracy for AGB during the B and EF stages, with R^2^ = 0.71 and 0.41, and rRMSE = 20.52% and 19.28%, respectively. The accuracy of the AGB estimation model based on ND TFs fell between the best and worst accuracy across the three window sizes.

### 3.4 Importance analysis of texture features for rice AGB estimation

**Figure 5:**
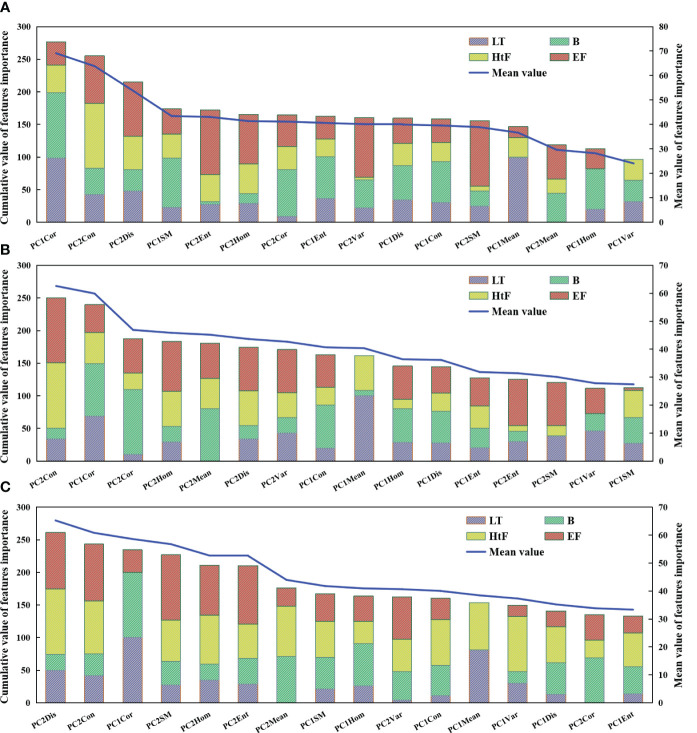
Importance analysis of texture features for rice AGB monitoring under small window size **(A)**, middle window size **(B)**, and large window size **(C)**. LT, Late Tillering stage; B, Booting stage; HtF, Heading to Flowering stage; EF, Early Filling stage.

In this study, we evaluated the importance scores of TFs at different growth stages in the D2 direction based on the RF models(**Figure 5**). The importance scores of TFs varied with window sizes. For small window size, the important TFs were PC1Cor, PC2Con, and PC2Dis. For medium window size, PC2Con and Cor were found to be important TFs. For large window size, PC2Dis, PC2Con, and PC1Cor were identified as significant TFs. The Hom, Ent, Cor, and Var from the PC2 image demonstrated a moderate level of importance scores.

**Figure 6:**
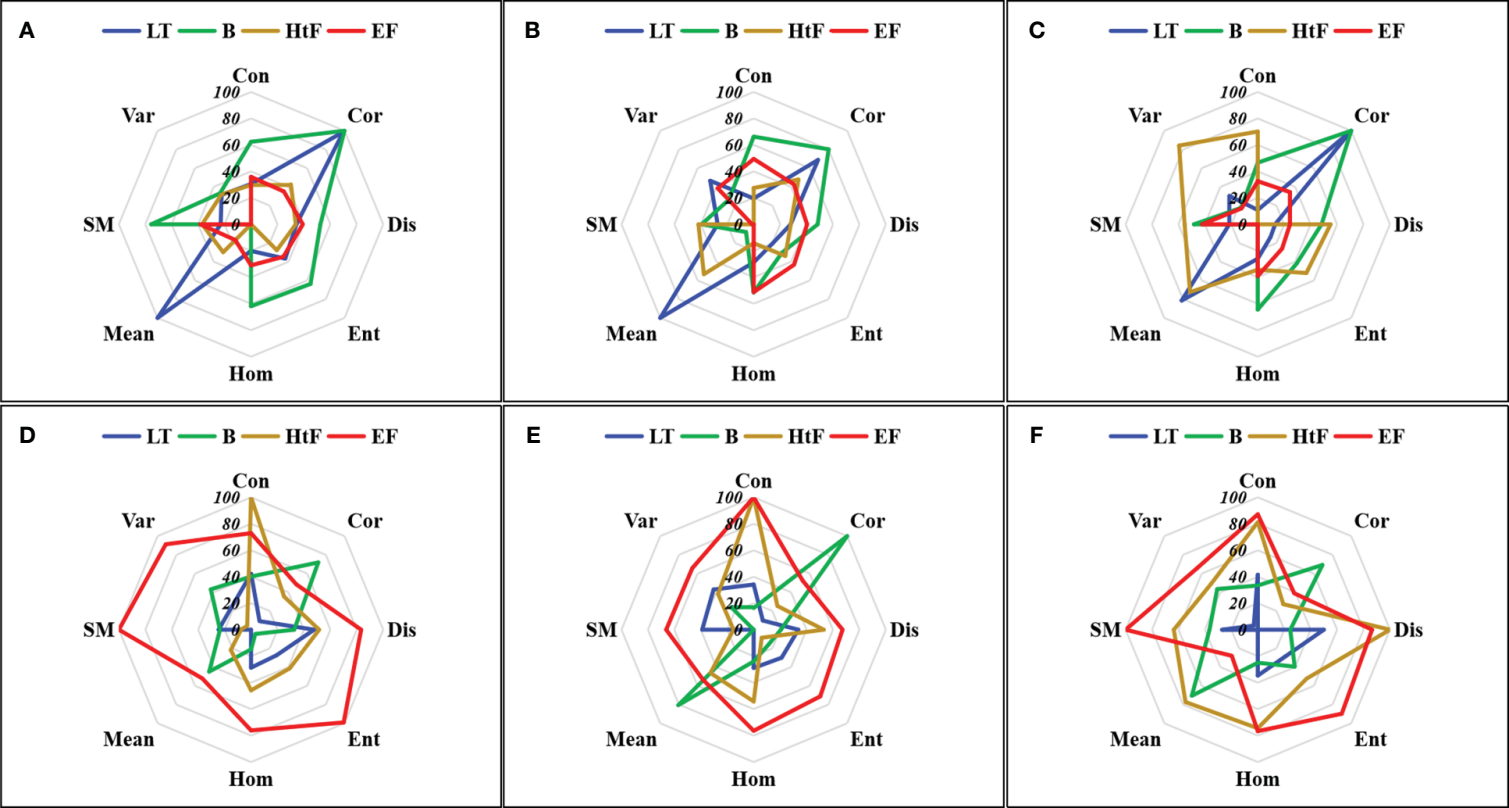
Importance analysis of texture features in the first principal component image **(A–C)** and second principal component image **(D–F)**. **(A, D)** Small window size; **(B, E)** Middle window size, and **(C, F)** Large window size. LT, Late Tillering stage; B, Booting stage; HtF, Heading to Flowering stage; EF, Early Filling stage.

Further analysis of the TFs extracted from different principal component images revealed an inverse variation in the importance scores of TFs between PC1 and PC2 images (**Figure 6**). During the vegetative growth period, the significance of TFs in the PC1 image exceeded that of the PC2 image. However, the TFs in the PC2 image were more prominent during the reproductive growth period. For the PC1 image, the TFs with higher importance scores were Mean, Cor, Con, and Hom. For the PC2 image, the TFs that stand out were SM, Ent, Con, Dis, and Hom.

The importance scores of TFs vary with the growth stages (**Figures 5**, **6**). At the LT stage, TFs such as Cor and Mean derived from the PC1 imagery demonstrated higher significance. Almost all TFs extracted from the B stage were found to be important across three different window sizes, and the most important TFs was Cor. At the HtF stage, the importance scores of Con, Dis, and Hom were greater than that of other TFs. At the EF stage, multiple TFs from the PC2 images show importance, such as SM, Ent, Hom, Dis, and Con.

**Figure 7:**
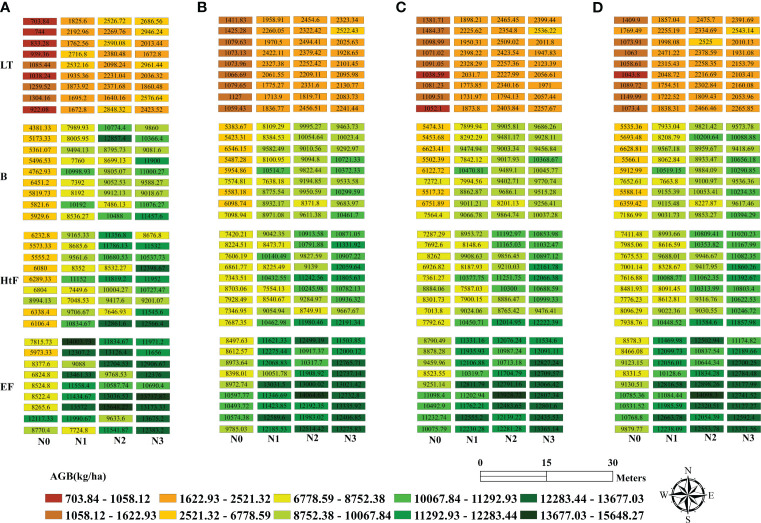
Rice AGB mapping based on field measurement **(A)** and estimated AGB values with texture features derived from D2 direction (45°); **(B)** Small window size; **(C)** Middle window size, and **(D)** Large window size. LT, Late Tillering stage; B, Booting stage; HtF, Heading to Flowering stage; EF, Early Filling stage.

**Figure 7** shows the spatial distribution of estimated rice AGB at various growth stages based on TFs derived from the D2 direction. The selection of the D2 direction allowed for a more effective assessment of the estimated accuracy in rice AGB throughout the growth stages, and more details can be found in the Results section (Section 3.3). The results demonstrated a high spatial consistency with the observed AGB throughout the entire growth season. AGB continued to increase, and the differences within subplots intensified over time. AGB showed correlations with nitrogen fertilization levels and rice varieties. As nitrogen application increased, AGB values also increased. Additionally, differences were observed among varieties, with V1 often exhibiting higher AGB values compared to V2 and V3.

### 3.5 Comparison of spectral-based and texture-based features for AGB estimation

This study also compared the differences in rice AGB estimation between TFs derived from optimal texture parameters and spectral features (SFs) (reflectance of spectral bands) (**Table 4**). At the LT stage, both SFs and TFs exhibited comparable accuracy for estimating AGB (TFs: R^2^=0.82, SFs: R^2^=0.82). During the mid-growth stages (e.g. B stage and HtF stage in this study), TFs tend to exhibit higher accuracy in AGB estimation compared to SFs (TFs: R^2^=0.83, SFs: R^2^=0.78 at the B stage, and TFs: R^2^=0.70, SFs: R^2^=0.47 at the HtF stage). At the EF stage, the accuracy of estimating AGB using TFs was lower compared to SFs (TFs: R^2^=0.58, SFs: R^2^=0.63). It is noteworthy that the fusion of SFs and TFs does not yield improved accuracy for rice AGB estimation compared to the use of either SFs or TFs alone (**Supplementary Table 5**).

**Table 4:** Estimation accuracy of rice AGB model during the critical growth stages based on spectral features.

Stages	Calibration set			Validation set		
	RMSE (kg/ha)	R^2^	rRMSE (%)	RMSE (kg/ha)	R^2^	rRMSE (%)
**LT**	237.56	0.89	12.21	343.95	0.82	18.44
**B**	779.01	0.90	9.09	1339.28	0.78	15.55
**HtF**	827.80	0.87	8.65	1931.32	0.47	22.62
**EF**	1021.92	0.87	8.77	1857.75	0.63	18.37
**All**	755.29	0.97	9.96	1938.27	0.79	23.96

## 4 Discussion

### 4.1 The optimal window size for extracting texture features

**Figure 8:**
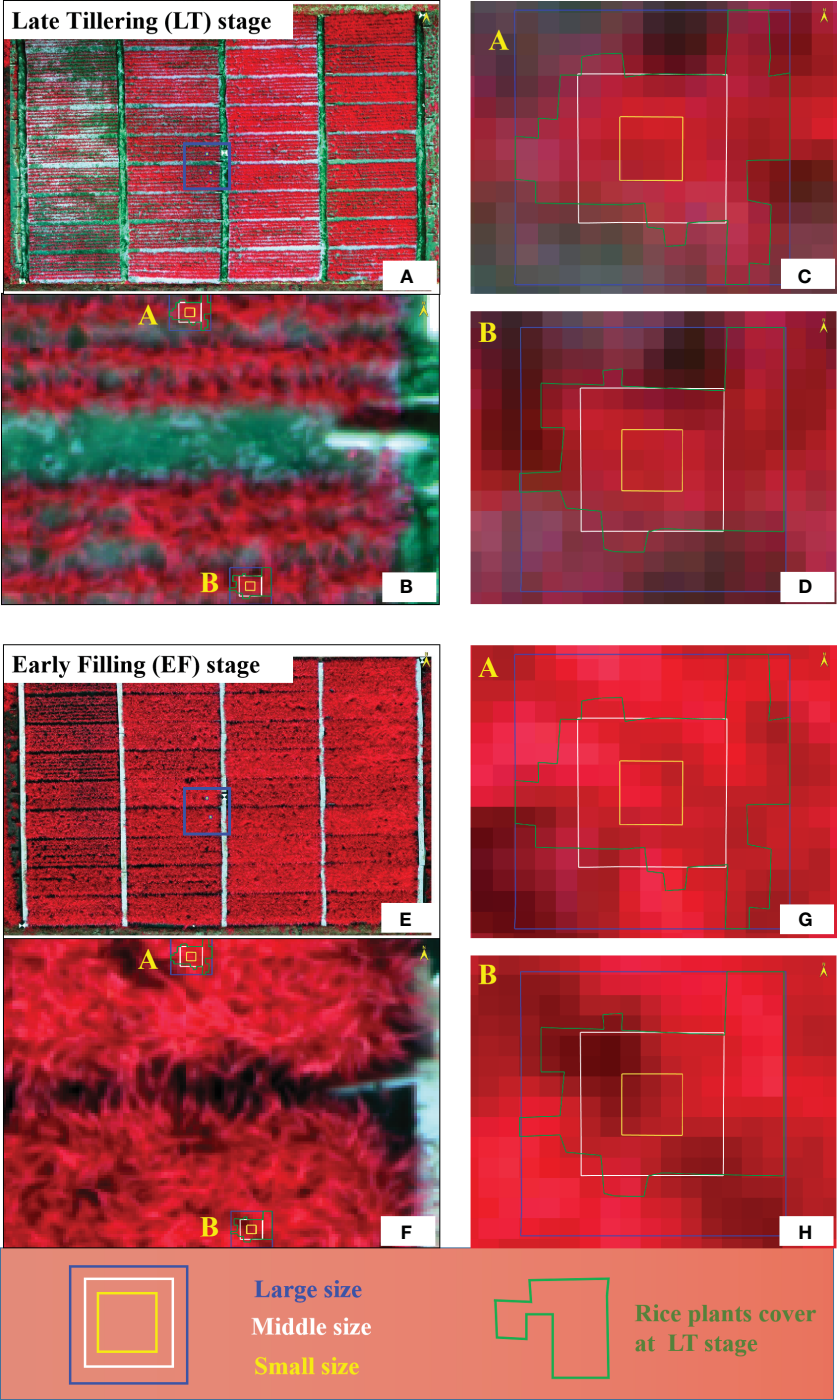
Dynamic changes of rice plant coverage with growth stage under different texture window sizes. The uppercase letters **(A, B)** represent two analysis points where the details within the respective window scales are examined at different magnification levels. **(A, E)** represent the full-view window at LT stage and EF stage, respectively; **(B, F)** represent the view window enlarged by 500%; **(C, D, G, H)** represent the view window enlarged by 2000%.

Window size is a crucial variable in TFs extraction, as different window sizes impact the frequency of pixel value occurrences during the process of texture calculation (Marceau et al., 1990; Liang et al., 2022; Liu et al., 2022a). To capture the object-specific TFs in an image, the window size must be smaller than the object’s size but large enough to include the variability of the features of the object (Rodriguez-Galiano et al., 2012; Zhou et al., 2017; Zhang et al., 2020; Liang et al., 2022). This study analyzed the relationship between TFs and AGB estimation accuracy under different window sizes and found that the optimal window size for computing TFs was closely related to the crop growth period (**Table 3**). During the vegetative growth stage, a small window size is more suitable, while the large window size appears to offer advantages in capturing TFs during the reproductive growth stage. The reason could be explained by the relative relationship between the rice canopy cover of different growth stage and window size. In general, at the early stage of rice growth (e.g., LT stage, **Figures 8A–D**), the rice plant were relatively small, and the canopy was partly closed. Small window size primarily captured the green information of rice plant, while minimizing the influence of water-soil background noise. This scale provided a more precise representation of the plant’s growth status and improved the AGB estimation. However, when using the large window size that exceeded the scope of the rice canopy, significant interference related to water-soil background noise occurred. This led to a decrease in the signal-to-noise ratio of the TFs, reducing the AGB estimation accuracy. At the mid-to-late stage of rice growth (e.g., EF stage, **Figures 8E–H**), the rice plant experienced vigorous growth, resulting in the formation of a dense canopy. At a small window scale, which mainly comprised panicles and some leaf organs, the TFs were insufficient to capture the macroscopic characteristics of rice plant. However, using a large window size that covers the entire canopy of rice plant, with minimal inclusion of background soil and water information, a more comprehensive depiction of plant structure is achieved. This scale allowed a more comprehensive characterization of the rice canopy, which increased the accuracy of AGB monitoring. This provided a brief explanation of the findings described in Result 3.2, which matched the findings of Yue et al. (2019) and Zhou et al. (2017).

Given the growth of rice plant, there exists a specific period during which the extent of rice plant coverage aligns with a window of moderate size. The medium-sized window was expected to yield the highest accuracy in AGB monitoring during this particular stage. However, we did not observe this outcome during our four growth stages, mainly because the sampling periods did not encompass this critical growth stage. We hypothesized that there might be an offsetting effect between the optimal texture window scales during the early and late stages of rice growth. As a result, the medium-sized window would provide the optimal AGB monitoring accuracy throughout the entire growth season. To further validate this hypothesis, we conducted RF model using all data collected throughout the growth season (**Table 5**); as anticipated, it has been found that the best accuracy in monitoring AGB can be achieved through the extraction of TFs at the intermediate window scale. Therefore, we suggested that it is imperative to consider using the appropriate window sizes for calculating texture parameters at different growth stages, particularly when utilizing these parameters for rice AGB estimation.

**Table 5:** Estimation results of rice AGB during the growth season based on texture features.

Windows	Directions	Calibration set			Validation set		
		RMSE (kg/ha)	R^2^	rRMSE (%)	RMSE (kg/ha)	R^2^	rRMSE (%)
**Small**	ND	624.4	0.98	8.23	2077.88	0.76	25.68
	D1	667.89	0.97	8.81	1956.36	0.79	24.18
	D2	560.70	0.99	7.39	1838.56	0.81	22.73
	D3	666.55	0.97	8.79	2162.41	0.74	26.73
	D4	686.44	0.97	9.05	1949.04	0.79	24.09
**Middle**	ND	565.31	0.98	7.45	1766.42	0.82	21.83
	D1	529.04	0.98	6.98	1746.74	0.83	21.59
	D2	565.59	0.98	7.46	1704.28	0.84	21.07
	D3	596.49	0.98	7.87	1769.39	0.83	21.87
	D4	596.03	0.98	7.86	1836.60	0.81	22.70
**Large**	ND	572.97	0.98	7.56	1758.00	0.83	21.73
	D1	624.10	0.98	8.23	1825.55	0.81	22.56
	D2	573.87	0.98	7.57	1711.52	0.84	21.16
	D3	611.56	0.98	8.06	1796.42	0.82	22.20
	D4	593.81	0.98	7.83	1823.08	0.81	22.53

### 4.2 The optimal direction parameter for extracting texture features

As one of the crucial research questions, the influence of direction on AGB monitoring is multifaceted and warrants thorough investigation (Hall-Beyer, 2007; Zheng et al., 2020; Liang et al., 2022; Liu et al., 2022a). Our findings suggested that D2 was the optimal direction for TFs extraction (**Table 3**), which matched the conclusions of Fu et al. (2020) and Fu et al. (2021). However, no definitive results have been obtained regarding the worst direction for TFs computations in this study. As we all know, rice plant naturally expands their growth in confined spaces; the planting distance between individual plants was smaller than the spacing between rows. Thus, they tended to close the spaces along the rows at the early growth stage, whereas, in the direction perpendicular to the planting rows, the plants closed the spaces during the middle period of growth. The diagonal directions (D2 and D4), which encompass both lateral and row-wise growth, offered a comprehensive reflection of the rice canopy distribution, making them more widely applicable for monitoring rice AGB. Along the planting rows, rice plant grow close together, reducing the influence of soil-water background noise. Even with the larger window size, canopy closes up in the early growth stage along the rows, and the pixel values become more similar, leading to decreased possibility of capturing spatial variations in rice canopy using TFs (Guo et al., 2021; Luo et al., 2022). The direction perpendicular to the planting rows (D3) is susceptible to background noise, significantly reducing the accuracy of AGB monitoring (Zheng et al., 2018; Zheng et al., 2020). The observed differences between the D2 and D4 directions may be attributed to the geometric relationship of the sun, sensor, and rice plant. We conducted all flights before noon (local time) when the sun was positioned in the southeast direction. The flight routes were perpendicular to the rows of rice plant, from northwest to southeast. Therefore, the D2 direction was closer to the backward observation, which led to consistently higher and brighter values were observed. This finding aligns with the conclusion drawn by Liang et al. (2022), who summarized that the most accurate estimation of rubber plantation AGB using TFs were achieved when conducting flight operations in the afternoon(local time) with 135° direction. We proposed this hypothesis to capture the interest of other researchers and encourage further investigation.

To further demonstrated the advantages of calculating TFs in the D2 direction, we conducted unified modeling throughout the whole growth season (**Table 5**). The results consistently showed that the D2 direction had the highest accuracy in AGB estimation, irrespective of the window size. This provided evidence supporting the rationale for considering the D2 direction as the optimal direction. Contrary to our finding, Zheng et al. (2020) found that texture information computed along the planting row direction (D1) is more advantageous for estimating rice leaf nitrogen content (LNC) and plant nitrogen content (PNC). This can be attributed to the lower imagery spatial resolution (5.4cm) in Zheng et al.’s study, they extracted TFs from 3 × 3 window size (approximately 16cm × 16cm), which primarily exhibit information pertaining to the row direction. Luo et al. (2022) suggested that TFs computed perpendicular to the ridges exhibited higher accuracy in estimating potato AGB. In contrast, Liu et al. (2022a) demonstrated that the direction selection did not affect the accuracy of TFs-based potato AGB estimation. The differences between these studies can be comprehensively considered from the perspective of image spatial resolution, crop type, planting pattern, and fertilizer application. Particularly, the significant impact of different spatial resolutions on GLCM-based TFs has been confirmed by previous studies (Yue et al., 2019; Liu et al., 2022a).

### 4.3 Important texture features for estimating rice AGB

**Figure 9:**
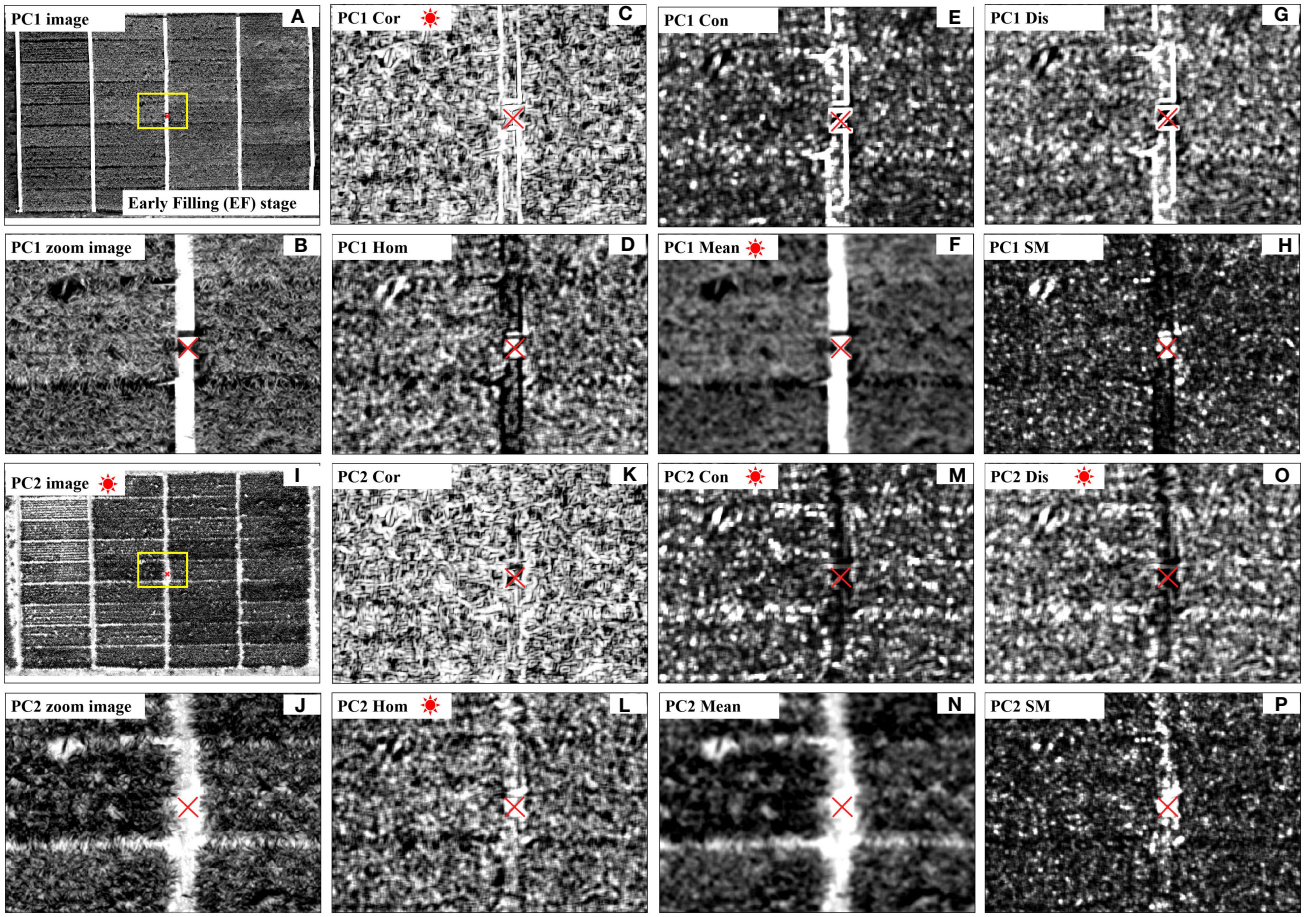
Important texture features for rice AGB estimation. The texture metrics Cor **(C, K)**, Hom **(D, L)**, Con **(E, M)**, Mean **(F, N)**, Dis **(G, O)** and SM **(H, P)** are from the principal component images; The PC1 **(A, B)** and PC2 **(I, J)** represent the first principal component image (PC1) and second principal component image (PC2); The red asterisks represent important texture features for rice AGB estimation, and the red multiplication symbol represents an important directional field object.

Although the tremendous potential of TFs in estimating crop growth parameters has been repeatedly demonstrated, there has been limited research on the physical meanings of these important features (Zheng et al., 2018; Liu C et al., 2019; Yang et al., 2019; Guo et al., 2021; Liu et al., 2022a). In light of this, this study focused on analyzing the mechanistic of TFs that are of significant value for rice AGB estimation (**Figure 9**).

Compared to the PC1 image, the PC2 image expresses more edge information of internal organs within the rice plant, between rice plant, and between rice and background (**Figures 9A, J**). It represents high-frequency information in the image and can better capture structural difference in the field. Therefore, TFs derived from the PC2 image have higher importance for rice AGB estimation compared to the TFs derived from the PC1 image (**Figure 6**). The importance analysis of TFs has identified three texture metrics that are consistently important throughout the entire growth period: PC1Cor, PC2Con, and PC2Dis (**Figure 5**). From the calculation form, it can be observed that Cor does not assign weights to the difference in digital numbers (DN) between neighboring pixels (**Table 2**). Unlike other TFs, Cor focuses on internal texture (Hall-Beyer, 2007; Hall-Beyer, 2017), which is consistent with the fact that PC1 image primarily reflects internal information within the plants or the background (**Figures 9A, B**). Indeed, internal texture primarily represents low-frequency information within a specific window, reflecting gradual changes within the rice plant (**Figure 9C**). It focuses on capturing information within the plants rather than between plant organs or between plants and background. Cor represents the vigorously growing portions of the rice plant, closely correlated with high AGB values. As for PC1Mean, a metric indicating internal uniformity (**Figure 9F**), has the same physical meaning as PC1Cor. These findings align consistently with the conclusions from Xu T. et al. (2022); Zhou L. et al. (2023), and Zhu et al. (2022). Unlike Cor and Mean, Con and Dis were utilized to enhance the edge information in the image (**Figures 9N, P**) (Haralick et al., 1973; Hall-Beyer, 2007; Hall-Beyer, 2017; Guo et al., 2021), indicating high-frequency information within the specific window, that is consistent with the PC2 image representing edge information. Both Con and Dis precisely described the relationship between pixel frequency and the distance from the diagonal, thereby enhancing the effectiveness of AGB estimation. These findings align with the results from Guo et al. (2021), where the performance of Con outperformed other TFs.

Similar to Cor, HOM is a metric that quantifies the homogeneity of the grey level in the GLCM window. The HOM calculated from the PC1 image reflects the uniformity of rice growth. Higher HOM values indicate more uniform rice growth, which is typically associated with higher AGB, consistent with the findings in previous study (Wang F. et al., 2021). The HOM calculated from the PC2 image reflects the relatively smooth portion of high-frequency information, which represents the information on rice plant or the soil background in mixed pixels. Studies by Wang W. et al. (2021) and Liu S. et al. (2023) respectively demonstrate that adjusted vegetation indices considering rice green plants abundance information or soil background in mixed pixel, such as adjusted abundance vegetation index (AAVI), and adjusted vegetation indices considering soil background (VI_CS_), contribute to improving the estimation accuracy of key crop growth parameters. This could be a potential reason for the importance scores of PC2HOM. However, this conclusion has not been confirmed for AGB estimation and will be one of our future research endeavors. SM shares similar physical implications with HOM, while Ent exhibits contrasting characteristics to HOM. Hence, both SM and Ent attain significant importance scores in estimating rice AGB.

### 4.4 Difference for rice AGB estimation between SFs and TFs

SFs derived from UAVs-based multispectral images are widely used in crop biophysical parameters estimation (Han et al., 2019; Maimaitijiang et al., 2019; Wang F. et al., 2022; Wang et al., 2023). This study compared the RF models’ performance between SFs and TFs for estimating the rice AGB (**Tables 3**–**5**). The result showed that the different performance between SFs and TFs was stage-specific. During the early stages of crop growth, the high leaf-stem density plays a significant role in determining a substantial portion of the AGB (Zhu et al., 2023). The SFs and TFs can reflect the reflectance attribute and spatial variation of rice canopy well, and the correlation between them is high (Bai et al., 2021). As a result, this explains the comparable relationship between these features when estimating AGB. For the mid-growth stages, the complex nature of the canopy during this stage makes it challenging for SFs alone to capture the spatial variability and intricate details, whereas TFs excel in capturing these fine-grained characteristics (Liu Y. et al., 2019; Yue et al., 2019; Zhang et al., 2021). Consequently, the superior performance of TFs in estimating AGB is observed during the mid-growth stages, particularly during the HtF stage. At the EF stage, the dense rice canopy imposes certain limitations on the representation of spatial heterogeneity by TFs, even in the presence of large windows.

However, in contrast to previous findings (Dube and Mutanga, 2015; Liu et al., 2018; Liang et al., 2022; Xu L. et al., 2022), the fused features did not exhibit superiority in estimating AGB, which may be attributed to that this study exclusively utilized band reflectance instead of vegetation indices, information overlap occurred between spectral reflectance and TFs, resulting in limited improvement in the predictive accuracy of rice AGB through feature fusion, which matched the findings of Mao et al. (2021). In addition, variations in the results can occur due to different crop types and regression algorithms used (Liu et al., 2018; Liang et al., 2022; Liu et al., 2022b; Xu L. et al., 2022; Liu Y. et al., 2023).

### 4.5 Limitations and directions of future work

Our findings provided compelling evidence of the influence of texture window size and direction on the rice AGB estimation using GLCM-based TFs. However, it is crucial to further validate its general applicability in terms of spatial and temporal transferability. In the future, the impact of texture parameters on crop AGB estimation still needs to be tested across diverse ecological conditions, multiple rice genotypes, and various crop types. Compared to high-resolution RGB images, multispectral imagery generally has lower spatial resolution (Lu et al., 2019; Liang et al., 2022; Gan et al., 2023). Therefore, further research is needed to investigate whether the conclusions based on multispectral imagery also apply to high-resolution RGB images.

Although the RF algorithm has strong regression capabilities (Liu Y. et al., 2019; Burdett and Wellen, 2022; Borrmann et al., 2023), this study has highlighted the potential issues of overfitting when dealing with small sample size. In order to improve the reliability of the RF model, it is essential to conduct additional field experiments across various locations, years, and crop varieties for further evaluation. Surprisingly, unlike previous studies, the incorporation of fused features did not notably improve the accuracy of AGB estimation (Dube and Mutanga, 2015; Liu et al., 2018; Liang et al., 2022; Xu L. et al., 2022). In addressing this issue in future research, two potential approaches can be considered. Firstly, before training the estimation models, an analysis can be conducted to assess the correlation among the input predictor variables, utilizing techniques such as Variance Inflation Factor (VIF) or feature selection methods. Secondly, the inclusion of modeling techniques with non-linear structures, such as Support Vector Regression (SVR), Extreme Learning Machines (ELM), and XGBoost, can be explored extensively to evaluate their impact on the estimation results.

## 5 Conclusions

Accurately assessment of AGB could provide valuable insights into the estimation and management of crop health and productivity. To resolve the saturation issue of spectral information, TFs were introduced to bridge this gap. This study provided a comprehensive evaluation of how GLCM-based TFs with different window size and direction parameters influence the accuracy of rice AGB estimation. The findings revealed that the appropriate window size for extracting TFs varies according to the rice growth stage, highlighting the need to incorporate multi-scale texture to capture the spatial variations of the rice canopy throughout the growing season. Additionally, the diagonal direction at 45° (D2) was identified as the optimal direction for estimating AGB. The important features of rice AGB estimation were Con, Dis, and Cor, which are mainly derived from the PC2 principal component image, which can better capture edge information. TFs were served as a valuable alternative or complement to spectral features, demonstrating estimation accuracy comparable to spectral reflectance for rice AGB estimation. These findings might help identify the best configuration of GLCM parameters to enhance the accuracy of estimating AGB, which can provide valuable insights for efficient monitoring of crop information in precision agriculture.

## Data availability statement

The original contributions presented in the study are included in the article/**Supplementary Material**. Further inquiries can be directed to the corresponding authors.

## Author contributions

JLiu: Conceptualization, Data curation, Formal analysis, Methodology, Validation, Writing – original draft, Writing – review & editing. YZ: Data curation, Formal analysis, Funding acquisition, Investigation, Methodology, Software, Visualization, Writing – original draft, Writing – review & editing. LS: Funding acquisition, Methodology, Project administration, Software, Supervision, Writing – review & editing. XS: Formal analysis, Investigation, Visualization, Writing – review & editing. JLi: Formal analysis, Investigation, Writing – review & editing. JZ: Formal analysis, Software, Writing – review & editing. XZ: Investigation, Writing – review & editing. LR: Formal analysis, Writing – review & editing. WW: Conceptualization, Formal analysis, Funding acquisition, Methodology, Supervision, Writing – review & editing. XL: Conceptualization, Funding acquisition, Investigation, Project administration, Supervision, Writing – review & editing.

## References

[B1] Alebele Y. Zhang X. Wang W. Yang G. Yao X. Zheng H.. (2020). Estimation of canopy biomass components in paddy rice from combined optical and sar data using multi-target gaussian regressor stacking. Remote Sensing. 12, 2564. doi: 10.3390/rs12162564

[B2] Bai X. Chen Y. Chen J. Cui W. Tai X. Zhang Z.. (2021). Optimal window size selection for spectral information extraction of sampling points from UAV multispectral images for soil moisture content inversion. Comput. Electron. Agriculture. 190, 106456. doi: 10.1016/j.compag.2021.106456

[B3] Bao Y. Gao W. Gao Z. (2009). Estimation of winter wheat biomass based on remote sensing data at various spatial and spectral resolutions. Front. Earth Sci. China. 3, 118–128. doi: 10.1007/s11707-009-0012-x

[B4] Borrmann P. Brandt P. Gerighausen H. (2023). Mispel: a multi-crop spectral library for statistical crop trait retrieval and agricultural monitoring. Remote Sensing. 15, 3664. doi: 10.3390/rs15143664

[B5] Breiman L. (2001). Random forests. Mach. Learning. 45, 5–32. doi: 10.1023/A:1010933404324

[B6] Burdett H. Wellen C. (2022). Statistical and machine learning methods for crop yield prediction in the context of precision agriculture. Precis. Agriculture. 23, 1553–1574. doi: 10.1007/s11119-022-09897-0

[B7] Cao J. Zhang Z. Tao F. Zhang L. Luo Y. Zhang J.. (2021). Integrating multi-source data for rice yield prediction across China using machine learning and deep learning approaches. Agric. For. Meteorology. 297, 108275. doi: 10.1016/j.agrformet.2020.108275

[B8] Cheng T. Song R. Li D. Zhou K. Zheng H. Yao X.. (2017). Spectroscopic estimation of biomass in canopy components of paddy rice using dry matter and chlorophyll indices. Remote Sensing. 9, 319. doi: 10.3390/rs9040319

[B9] Cui Z. Kerekes J. P. (2018a). Potential of red edge spectral bands in future landsat satellites on agroecosystem canopy green leaf area index retrieval. Remote Sensing. 10 (9), 1458. doi: 10.3390/rs10091458

[B10] Cui Z. Kerekes J. P. (2018b). Impact of wavelength shift in relative spectral response at high angluaves of incidence in landsat-8 operational land imager and future landsat design concepts. IEEE Trans. Geosci. Remote Sensing. 56, 5873–5883. doi: 10.1109/tgrs.2018.2827394

[B11] Di Gennaro S. F. Toscano P. Gatti M. Poni S. Berton A. Matese A. (2022). Spectral comparison of -based hyper and multispectral cameras for precision viticulture. Remote Sensing. 14, 449. doi: 10.3390/rs14030449

[B12] Dube T. Mutanga O. (2015). Investigating the robustness of the new landsat-8 operational land imager derived texture metrics in estimating plantation forest aboveground biomass in resource constrained areas. ISPRS J. Photogrammetry Remote Sensing. 108, 12–32. doi: 10.1016/j.isprsjprs.2015.06.002

[B13] Fu Y. Yang G. Li Z. Song X. Li Z. Xu X. (2020). Winter Wheat Nitrogen Status Estimation Using UAV-Based RGB Imagery and Gaussian Processes Regression. Remote Sens. 12, 3778. doi: 10.3390/rs12223778

[B14] Fu Y. Yang G. Song X. Li Z. Xu X. Feng H.. (2021). Improved estimation of winter wheat aboveground biomass using multiscale textures extracted from UAV-based digital images and hyperspectral feature analysis. Remote Sensing. 13, 581. doi: 10.3390/rs13040581

[B15] Fu Y. Yang G. Wang J. Song X. Feng H. (2014). Winter wheat biomass estimation based on spectral indices, band depth analysis and partial least squares regression using hyperspectral measurements. Comput. Electron. Agriculture. 100, 51–59. doi: 10.1016/j.compag.2013.10.010

[B16] Gan Y. Wang Q. Matsuzawa T. Song G. Iio A. (2023). Multivariate regressions coupling colorimetric and textural features derived from UAV-based rgb images can trace spatiotemporal variations of lai well in a deciduous forest. Int. J. Remote Sensing. 44, 4559–4577. doi: 10.1080/01431161.2023.2208709

[B17] Gnyp M. L. Miao Y. Yuan F. Ustin S. L. Yu K. Yao Y.. (2014). Hyperspectral canopy sensing of paddy rice aboveground biomass at different growth stages. Field Crops Res. 155, 42–55. doi: 10.1016/j.fcr.2013.09.023

[B18] Guo Y. Fu Y. H. Chen S. Robin Bryant C. Li X. Senthilnath J.. (2021). Integrating spectral and textural information for identifying the tasseling date of summer maize using UAV based RGB images. Int. J. Appl. Earth Observation Geoinformation. 102, 102435. doi: 10.1016/j.jag.2021.102435

[B19] Hall-Beyer M. (2007) GLCM Texture: A Tutorial v. 1.0 through 2.7. Available at: http://www.fp.ucalgary.ca/mhallbey/tutorial.htm.

[B20] Hall-Beyer M. (2017). Practical guidelines for choosing glcm textures to use in landscape classification tasks over a range of moderate spatial scales. Int. J. Remote Sensing. 38, 1312–1338. doi: 10.1080/01431161.2016.1278314

[B21] Han L. Yang G. Dai H. Xu B. Yang H. Feng H.. (2019). Modeling maize above-ground biomass based on machine learning approaches using UAV remote-sensing data. Plant Methods 15, 1–19. doi: 10.1186/s13007-019-0394-z 30740136 PMC6360736

[B22] Haralick R. M. Shanmugam K. Dinstein I. H. (1973). Textural features for image classification. IEEE Trans. On Systems Man Cybernetics. 3, 610–621. doi: 10.1109/TSMC.1973.4309314

[B23] Jangra S. Chaudhary V. Yadav R. C. Yadav N. R. (2021). High-throughput phenotyping: a platform to accelerate crop improvement. Phenomics. 1, 31–53. doi: 10.1007/s43657-020-00007-6 36939738 PMC9590473

[B24] Jin X. Zarco-Tejada P. Schmidhalter U. Reynolds M. P. Hawkesford M. J. Varshney R. K.. (2020). High-throughput estimation of crop traits: a review of ground and aerial phenotyping platforms. IEEE Geosci. Remote Sens. Magazine 9 (1), 200–231. doi: 10.1109/MGRS.2020.2998816

[B25] Kelsey K. C. Neff J. C. (2014). Estimates of aboveground biomass from texture analysis of landsat imagery. Remote Sensing. 6, 6407–6422. doi: 10.3390/rs6076407

[B26] Lakhiar I. A. Jianmin G. Syed T. N. Chandio F. A. Buttar N. A. Qureshi W. A. (2018). Monitoring and control systems in agriculture using intelligent sensor techniques: a review of the aeroponic system. J. Sensors. 2018, 1–18. doi: 10.1155/2018/8672769

[B27] Li B. Xu X. Zhang L. Han J. Bian C. Li G.. (2020). Above-ground biomass estimation and yield prediction in potato by using UAV-based RGB and hyperspectral imaging. ISPRS J. Photogrammetry Remote Sensing. 162, 161–172. doi: 10.1016/j.isprsjprs.2020.02.013

[B28] Li S. Yuan F. Ata-UI-Karim S. T. Zheng H. Cheng T. Liu X.. (2019). Combining color indices and textures of UAV-based digital imagery for rice lai estimation. Remote Sensing. 11, 1763. doi: 10.3390/rs11151763

[B29] Li Z. Zhao Y. Taylor J. Gaulton R. Jin X. Song X.. (2022). Comparison and transferability of thermal, temporal and phenological-based in-season predictions of above-ground biomass in wheat crops from proximal crop reflectance data. Remote Sens. Environ. 273, 112967. doi: 10.1016/j.rse.2022.112967

[B30] Liang Y. Kou W. Lai H. Wang J. Wang Q. Xu W.. (2022). Improved estimation of aboveground biomass in rubber plantations by fusing spectral and textural information from UAV-based RGB imagery. Ecol. Indicators. 142, 109286. doi: 10.1016/j.ecolind.2022.109286

[B31] Liao C. Wang J. Dong T. Shang J. Liu J. Song Y. (2019). Using spatio-temporal fusion of landsat-8 and modis data to derive phenology, biomass and yield estimates for corn and soybean. Sci. Total Environment. 650, 1707–1721. doi: 10.1016/j.scitotenv.2018.09.308 30273730

[B32] Liu Y. Feng H. Yue J. Fan Y. Bian M. Ma Y.. (2023). Estimating potato above-ground biomass by using integrated unmanned aerial system-based optical, structural, and textural canopy measurements. Comput. Electron. Agriculture. 213, 108229. doi: 10.1016/j.compag.2023.108229

[B33] Liu Y. Feng H. Yue J. Jin X. Li Z. Yang G. (2022a). Estimation of potato above-ground biomass based on unmanned aerial vehicle red-green-blue images with different texture features and crop height. Front. Plant Science. 13. doi: 10.3389/fpls.2022.938216 PMC9452666 36092445

[B34] Liu Y. Feng H. Yue J. Li Z. Yang G. Song X.. (2022b). Remote-sensing estimation of potato above-ground biomass based on spectral and spatial features extracted from high-definition digital camera images. Comput. Electron. Agriculture. 198, 107089. doi: 10.1016/j.compag.2022.107089

[B35] Liu S. Jin X. Bai Y. Wu W. Cui N. Cheng M.. (2023). UAV multispectral images for accurate estimation of the maize LAI considering the effect of soil background. Int. J. Appl. Earth Observation Geoinformation. 121, 103383. doi: 10.1016/j.jag.2023.103383

[B36] Liu Y. Liu S. Li J. Guo X. Wang S. Lu J. (2019). Estimating biomass of winter oilseed rape using vegetation indices and texture metrics derived from UAV multispectral images. Comput. Electron. Agriculture. 166, 105026. doi: 10.1016/j.compag.2019.105026

[B37] Liu C. Yang G. Li Z. Tang F. Feng H. Wang J.. (2019). “Monitoring of winter wheat biomass using UAV hyperspectral texture features,” in. Li D. Zhao C. Computer and Computing Technologies in Agriculture XI. CCTA 2017. IFIP Advances in Information and Communication Technology, vol 546. Springer, Cham. doi: 10.1007/978-3-030-06179-1_25

[B38] Liu C. Yang G. Li Z. Tang F. Wang J. Zhang C.. (2018). Biomass estimation in winter wheat by UAV spectral information and texture information fusion. Scientia Agricultura Sin. 51, 3060–3073. doi: 10.3864/j.issn.0578-1752.2018.16.003

[B39] Liu J. Zhu Y. Tao X. Chen X. Li X. (2022). Rapid prediction of winter wheat yield and nitrogen use efficiency using consumer-grade unmanned aerial vehicles multispectral imagery. Front. Plant Sci. 13. doi: 10.3389/fpls.2022.1032170 PMC9638066 36352879

[B40] Lu D. (2005). Aboveground biomass estimation using landsat tm data in the Brazilian amazon. Int. J. Remote Sensing. 26, 2509–2525. doi: 10.1080/01431160500142145

[B41] Lu N. Zhou J. Han Z. Li D. Cao Q. Yao X.. (2019). Improved estimation of aboveground biomass in wheat from rgb imagery and point cloud data acquired with a low-cost unmanned aerial vehicle system. Plant Methods 15, 1–16. doi: 10.1186/s13007-019-0402-3 30828356 PMC6381699

[B42] Luo S. Jiang X. He Y. Li J. Jiao W. Zhang S.. (2022). Multi-dimensional variables and feature parameter selection for aboveground biomass estimation of potato based on UAV multispectral imagery. Front. Plant Science. 13. doi: 10.3389/fpls.2022.948249 PMC9372391 35968116

[B43] Maes W. H. Steppe K. (2019). Perspectives for remote sensing with unmanned aerial vehicles in precision agriculture. Trends Plant Science. 24, 152–164. doi: 10.1016/j.tplants.2018.11.007 30558964

[B44] Maimaitijiang M. Sagan V. Sidike P. Maimaitiyiming M. Hartling S. Peterson K. T.. (2019). Vegetation index weighted canopy volume model (CVMVI) for soybean biomass estimation from unmanned aerial system-based rgb imagery. ISPRS J. Photogrammetry Remote Sensing. 151, 27–41. doi: 10.1016/j.isprsjprs.2019.03.003

[B45] Mao P. Qin L. Hao M. Zhao W. Luo J. Qiu X.. (2021). An improved approach to estimate above-groundvolume and biomass of desert shrub communities based on UAV RGB images. Ecol. Indicators. 125, 107494. doi: 10.1016/j.ecolind.2021.107494

[B46] Marceau D. J. Howarth P. J. Dubois J. M. Gratton D. J. (1990). Evaluation of the grey-level co-occurrence matrix method for land-cover classification using spot imagery. IEEE Trans. On Geosci. Remote Sensing. 28, 513–519. doi: 10.1109/TGRS.1990.572937

[B47] Mercier A. Betbeder J. Baudry J. Le Roux V. Spicher F. Lacoux J.. (2020a). Evaluation of sentinel-1 & 2 time series for predicting wheat and rapeseed phenological stages. ISPRS J. Photogrammetry Remote Sensing. 163, 231–256. doi: 10.1016/j.isprsjprs.2020.03.009

[B48] Mercier A. Betbeder J. Rapinel S. Jegou N. Baudry J. Hubert-Moy L. (2020b). Evaluation of sentinel-1 and -2 time series for estimating lai and biomass of wheat and rapeseed crop types. J. Appl. Remote Sensing. 14, 1. doi: 10.1117/1.JRS.14.024512

[B49] Mukherjee A. Misra S. Raghuwanshi N. S. (2019). A survey of unmanned aerial sensing solutions in precision agriculture. J. Network Comput. Applications. 148, 102461. doi: 10.1016/j.jnca.2019.102461

[B50] Naidoo L. Main R. Cho M. A. Madonsela S. Majozi N. (2021). “Estimating South African maize biomass using integrated high-resolution UAV and sentinel 1 and 2 datasets,” 2021 IEEE International Geoscience and Remote Sensing Symposium IGARSS, Brussels, Belgium, 2021,1594–1596. doi: 10.1109/IGARSS47720.2021.9554261

[B51] Pacifici F. Chini M. Emery W. J. (2009). A neural network approach using multi-scale textural metrics from very high-resolution panchromatic imagery for urban land-use classification. Remote Sens. Environment. 113, 1276–1292. doi: 10.1016/j.rse.2009.02.014

[B52] Peng S. Tang Q. Zou Y. (2009). Current status and challenges of rice production in China. Plant Production Science. 12, 3–8. doi: 10.1626/pps.12.3

[B53] Qiu R. Wei S. Zhang M. Li H. Sun H. Liu G.. (2018). Sensors for measuring plant phenotyping: a review. Int. J. Agric. Biol. Engineering. 11, 1–17. doi: 10.25165/j.ijabe.20181102.2696

[B54] Richter K. Atzberger C. Hank T. B. Mauser W. (2012). Derivation of biophysical variables from earth observation data: validation and statistical measures. J. Appl. Remote Sensing. 6, 63557. doi: 10.1117/1.JRS.6.063557

[B55] Rodriguez-Galiano V. F. Chica-Olmo M. Abarca-Hernandez F. Atkinson P. M. Jeganathan C. (2012). Random forest classification of mediterranean land cover using multi-seasonal imagery and multi-seasonal texture. Remote Sens. Environment. 121, 93–107. doi: 10.1016/j.rse.2011.12.003

[B56] Seck P. A. Diagne A. Mohanty S. Wopereis M. C. (2012). Crops that feed the world 7: rice. Food Secur. 4, 7–24. doi: 10.1007/s12571-012-0168-1

[B57] Spiertz J. H. J. Ewert F. (2009). Crop production and resource use to meet the growing demand for food, feed and fuel: opportunities and constraints. Njas - Wageningen J. Life Sci. 56, 281–300. doi: 10.1016/S1573-5214(09)80001-8

[B58] Teng X. Dong Y. Meng L. (2015). “The study of winter wheat biomass estimation model based on hyperspectral remote sensing,” in Eds. Li D. Li Z. Computer and computing technologies in agriculture IX. CCTA 2015. IFIP advances in information and communication technology, vol. 479 (Springer, Cham). doi: 10.1007/978-3-319-48354-2_17

[B59] Wang D. Li R. Zhu B. Liu T. Sun C. Guo W. (2023). Estimation of wheat plant height and biomass by combining UAV imagery and elevation data. Agriculture. 13, 9. doi: 10.3390/agriculture13010009

[B60] Wang Q. Putri N. A. Gan Y. Song G. (2022). Combining both spectral and textural indices for alleviating saturation problem in forest lai estimation using sentinel-2 data. Geocarto Int. 37, 10511–10531. doi: 10.1080/10106049.2022.2037730

[B61] Wang W. Wu Y. Zhang Q. Zheng H. Yao X. Zhu Y.. (2021). AAVI: A novel approach to estimating leaf nitrogen concentration in rice from unmanned aerial vehicle multispectral imagery at early and middle growth stages. IEEE J. Selected Topics Appl. Earth Observations Remote Sensing. 14, 6716–6728. doi: 10.1109/JSTARS.2021.3086580

[B62] Wang F. Yang M. Ma L. Zhang T. Qin W. Li W.. (2022). Estimation of above-ground biomass of winter wheat based on consumer-grade multi-spectral UAV. Remote Sensing. 14, 1251. doi: 10.3390/rs14051251

[B63] Wang F. Yi Q. Hu J. Xie L. Yao X. Xu T.. (2021). Combining spectral and textural information in UAV hyperspectral images to estimate rice grain yield. Int. J. Appl. Earth Observation Geoinformation. 102, 102397. doi: 10.1016/j.jag.2021.102397

[B64] Wang W. Zheng H. Wu Y. Yao X. Zhu Y. Cao W.. (2022). An assessment of background removal approaches for improved estimation of rice leaf nitrogen concentration with unmanned aerial vehicle multispectral imagery at various observation times. Field Crops Res. 283, 108543. doi: 10.1016/j.fcr.2022.108543

[B65] Wang L. Zhou X. Zhu X. Dong Z. Guo W. (2016). Estimation of biomass in wheat using random forest regression algorithm and remote sensing data. Crop J. 4, 212–219. doi: 10.1016/j.cj.2016.01.008

[B66] Xu T. Wang F. Xie L. Yao X. Zheng J. Li J.. (2022). Integrating the textural and spectral information of UAV hyperspectral images for the improved estimation of rice aboveground biomass. Remote Sensing. 14, 2534. doi: 10.3390/rs14112534

[B67] Xu L. Zhou L. Meng R. Zhao F. Lv Z. Xu B.. (2022). An improved approach to estimate ratoon rice aboveground biomass by integrating UAV-based spectral, textural and structural features. Precis. Agriculture. 23, 1276–1301. doi: 10.1007/s11119-022-09884-5

[B68] Yang J. Ding F. Chen C. Liu T. Sun C. Ding D.. (2019). Correlation of wheat biomass and yield with UAV image characteristic parameters. Trans. Chin. Soc. Agric. Engineering. 35, 104–110. doi: 10.11975/j.issn.1002-6819.2019.23.013

[B69] Yu N. Li L. Schmitz N. Tian L. F. Greenberg J. A. Diers B. W. (2016). Development of methods to improve soybean yield estimation and predict plant maturity with an unmanned aerial vehicle based platform. Remote Sens. Environment. 187, 91–101. doi: 10.1016/j.rse.2016.10.005

[B70] Yue J. Yang G. Tian Q. Feng H. Xu K. Zhou C. (2019). Estimate of winter-wheat above-ground biomass based on UAV ultrahigh-ground-resolution image textures and vegetation indices. ISPRS J. Photogrammetry Remote Sensing. 150, 226–244. doi: 10.1016/j.isprsjprs.2019.02.022

[B71] Zhang D. Y. Han X. X. Lin F. F. Du S. Z. Zhang G. Hong Q. (2022). Estimation of winter wheat leaf area index using multi-source UAV image feature fusion. Trans. Chin. Soc. Agric. Eng. 38, 171–179. doi: 10.11975/j.issn.1002-6819.2022.09.018

[B72] Zhang C. Huang C. Li H. Liu Q. Li J. Bridhikitti A.. (2020). Effect of textural features in remote sensed data on rubber plantation extraction at different levels of spatial resolution. Forests. 11, 399. doi: 10.3390/f11040399

[B73] Zhang J. Qiu X. Wu Y. Zhu Y. Cao Q. Liu X.. (2021). Combining texture, color, and vegetation indices from fixed-wing uas imagery to estimate wheat growth parameters using multivariate regression methods. Comput. Electron. Agriculture. 185, 106138. doi: 10.1016/j.compag.2021.106138

[B74] Zhang Y. Ta N. Guo S. Chen Q. Zhao L. Li F.. (2022). Combining spectral and textural information from UAV RGB images for leaf area index monitoring in kiwifruit orchard. Remote Sensing. 14, 1063. doi: 10.3390/rs14051063

[B75] Zheng H. Cheng T. Li D. Yao X. Tian Y. Cao W.. (2018). Combining unmanned aerial vehicle (UAV)-based multispectral imagery and ground-based hyperspectral data for plant nitrogen concentration estimation in rice. Front. Plant Sci. 9. doi: 10.3389/fpls.2018.00936 PMC6043795 30034405

[B76] Zheng H. Cheng T. Zhou M. Li D. Yao X. Tian Y.. (2019). Improved estimation of rice aboveground biomass combining textural and spectral analysis of UAV imagery. Precis. Agriculture. 20, 611–629. doi: 10.1007/s11119-018-9600-7

[B77] Zheng H. Ma J. Zhou M. Li D. Yao X. Cao W.. (2020). Enhancing the nitrogen signals of rice canopies across critical growth stages through the integration of textural and spectral information from unmanned aerial vehicle (UAV) multispectral imagery. Remote Sensing. 12, 957. doi: 10.3390/rs12060957

[B78] Zhou J. Yan G. Sun M. Di T. Wang S. Zhai J. Zhao Z. (2017). The effects of GLCM parameters on LAI estimation using texture values from Quickbird satellite imagery. Sci. Rep. 7, 7366. doi: 10.1038/s41598-017-07951-w 28779107 PMC5544764

[B79] Zhou L. Nie C. Su T. Xu X. Song Y. Yin D.. (2023). Evaluating the canopy chlorophyll density of maize at the whole growth stage based on multi-scale UAV image feature fusion and machine learning methods. Agriculture. 13, 895. doi: 10.3390/agriculture13040895

[B80] Zhou M. Zheng H. He C. Liu P. Awan G. M. Wang X.. (2023). Wheat phenology detection with the methodology of classification based on the time-series UAV images. Field Crops Res. 292, 108798. doi: 10.1016/j.fcr.2022.108798

[B81] Zhu Y. Liu J. Tao X. Su X. Li W. Zha H.. (2023). A three-dimensional conceptual model for estimating the above-ground biomass of winter wheat using digital and multispectral unmanned aerial vehicle images at various growth stages. Remote Sensing. 15, 3332. doi: 10.3390/rs15133332

[B82] Zhu W. Rezaei E. E. Nouri H. Sun Z. Li J. Yu D.. (2022). UAV-based indicators of crop growth are robust for distinct water and nutrient management but vary between crop development phases. Field Crops Res. 284, 108582. doi: 10.1016/j.fcr.2022.108582

