# Corrigendum: Optimizing window size and directional parameters of GLCM texture features for estimating rice AGB based on UAVs multispectral imagery

**DOI:** 10.3389/fpls.2024.1378628

**Published:** 2024-02-28

**Authors:** Jikai Liu, Yongji Zhu, Lijuan Song, Xiangxiang Su, Jun Li, Jing Zheng, Xueqing Zhu, Lantian Ren, Wenhui Wang, Xinwei Li

**Affiliations:** ^1^ College of Resource and Environment, Anhui Science and Technology University, Chuzhou, Anhui, China; ^2^ Anhui Province Crop Intelligent Planting and Processing Technology Engineering Research Center, Anhui Science and Technology University, Chuzhou, Anhui, China; ^3^ Institute of Agricultural Remote Sensing and Information, Heilongjiang Academy of Agricultural Sciences, Harbin, Heilongjiang, China; ^4^ School of Management, Heilongjiang University of Science and Technology, Harbin, Heilongjiang, China; ^5^ College of Life Science, Langfang Normal University, Langfang, Hebei, China; ^6^ College of Agriculture, Anhui Science and Technology University, Chuzhou, Anhui, China

**Keywords:** unmanned aerial vehicles (UAVs), aboveground biomass (AGB), multispectral imagery, texture features (TFs), grey level co-occurrence matrix (GLCM), rice

In the published article, there was an error in the UAVs data acquisition time of three rice phenological phase. **2 Materials and methods**, *2.3.1 UAVs data acquisition and preprocessing*, Paragraph 1 previously stated: “The DJI Phantom 4 Multispectral RTK (P4M) UAVs (DJI, Shenzhen, Guangdong, China) was used to acquire multispectral images at four growth stages, including the late tillering stage (LT: 25/07/2020), booting stage (B: 23/08/2023), heading to flowering stage (HtF: 31/08/2023), and early filling stage (EF: 09/09/2023) (Table 1).”

The corrected sentence appears below:

“The DJI Phantom 4 Multispectral RTK (P4M) UAVs (DJI, Shenzhen, Guangdong, China) was used to acquire multispectral images at four growth stages, including the late tillering stage (LT: 25/07/2020), booting stage (B: 23/08/2020), heading to flowering stage (HtF: 31/08/2020), and early filling stage (EF: 09/09/2020) (Table 1).”

The authors apologize for this error and state that this does not change the scientific conclusions of the article in any way. The original article has been updated.

