# Supplementary material for: Optimizing window size and directional parameters of GLCM texture features for estimating rice AGB based on UAVs multispectral imagery
Source: Front Plant Sci. 2023 Dec 19;14:1284235. doi: 10.3389/fpls.2023.1284235 (PMC10773816; doi:10.3389/fpls.2023.1284235)
Supplement: Supplementary file 1 [file DataSheet_1.zip › Supplementary material/Supplementary material.docx]

Optimizing window size and directional parameters of GLCM texture features for estimating rice AGB based on UAV multispectral imagery

Jikai Liu^1,2,†^, Yongji Zhu^1,†^, Lijuan Song^3,4^, Xiangxiang Su^1^, Jun Li^1^, Jing Zheng^5^, Xueqing Zhu^1^, Lantian Ren^2,6^, Wenhui Wang^5,*^, and Xinwei Li^1,2,*^

^1^College of Resource and Environment, Anhui Science and Technology University, Chuzhou, Anhui, China;

^2^Anhui Province Crop Intelligent Planting and Processing Technology Engineering Research Center, Anhui Science and Technology University, Chuzhou, Anhui, China

^3^Institute of Agricultural Remote Sensing and Information, Heilongjiang Academy of Agricultural Sciences, Harbin, Heilongjiang, China;

^4^School of Management, Heilongjiang University of Science and Technology, Harbin, Heilongjiang, China;

^5^College of Life Science, Langfang Normal University, Langfang, Hebei, China;

^6^College of Agriculture, Anhui Science and Technology University, Chuzhou, Anhui, China;

*** Correspondence:**Xinwei Li
[lixw@ahstu.edu.cn](mailto:lixw@ahstu.edu.cn)

Wenhui Wang
1172139@lfnu.edu.cn

† These authors have contributed equally to this work

**Supplementary material**

**TABLE 1.** DJI P4M camera technical specifications

| **Parameters** | **Values** |
| --- | --- |
| Sensor | 1/2.9 inch CMOS(5) |
| Pixel resolution(px*px) | 1600*1300 |
| Acquisition mode | snapshot |
| Optics | f/2.20 |
| Focal length(mm) | 5.74 |
| FOV(°) | 62.7 |
| Battery life(minutes) | 27 |
| RTK accuracy(m) | vertical:±0.1; horizontal:±0.1 |
| Photo format | .tiff |
| Bands set | Blue：450 nm ± 16 nm |
|  | Green：560 nm ± 16 nm |
|  | Red：650 nm ± 16 nm |
|  | Red Edge：730 nm ± 16 nm |
|  | NIR：840 nm ± 26 nm |

**TABLE 2.** Descriptive statistics for AGB (kg/ha) of calibration and validation datasets, SD=Standard Deviation; CV=Coefficient of Variation.

| **Stages** | **Datasets** | **Min** | **Max** | **Mean** | **SD** | **CV** |
| --- | --- | --- | --- | --- | --- | --- |
| LT | Calibration | 703.84 | 2946.24 | 1946.04 | 621.49 | 0.32 |
|  | Validation | 744.00 | 2961.44 | 1865.47 | 782.52 | 0.42 |
| B | Calibration | 4381.33 | 11900.00 | 8570.14 | 2077.63 | 0.24 |
|  | Validation | 5173.33 | 12857.60 | 8611.29 | 2612.27 | 0.30 |
| HtF | Calibration | 6080.00 | 12861.60 | 9570.96 | 2100.03 | 0.22 |
|  | Validation | 5555.20 | 11952.00 | 8536.24 | 2454.26 | 0.29 |
| EF | Calibration | 6824.80 | 15648.27 | 11649.81 | 2225.34 | 0.19 |
|  | Validation | 5973.33 | 13173.33 | 10111.47 | 2173.07 | 0.21 |
| All Satge | Calibration | 703.84 | 14003.73 | 7583.25 | 3918.43 | 0.52 |
|  | Validation | 833.28 | 15648.27 | 8090.36 | 4241.14 | 0.52 |

**TABLE 3.** Variation of rice AGB (kg/ha) with the growth stages, SD=Standard Deviation; CV=Coefficient of Variation.

| **Stages** | **Min** | **Max** | **Mean** | **SD** | **CV** |
| --- | --- | --- | --- | --- | --- |
| LT | 703.84 | 2961.44 | 1921.42 | 664.25 | 0.35 |
| B | 4381.33 | 12857.6 | 8582.72 | 2215.85 | 0.26 |
| HtF | 5555.2 | 12861.6 | 9254.80 | 2231.3 | 0.24 |
| EF | 5973.33 | 15648.27 | 11179.76 | 2293.79 | 0.21 |
| All | 703.84 | 15648.27 | 7734.67 | 4009.28 | 0.52 |

**TABLE 4.** Variation of rice AGB (kg/ha) with the N levels, SD=Standard Deviation; CV=Coefficient of Variation.

| **N levels** | **Min** | **Max** | **Mean** | **SD** | **CV** |
| --- | --- | --- | --- | --- | --- |
| N0 | 703.84 | 12117.33 | 5288.69 | 2898.64 | 0.55 |
| N1 | 1672.80 | 14003.73 | 7999.30 | 3919.40 | 0.49 |
| N2 | 1640.16 | 15648.27 | 8648.98 | 4057.15 | 0.47 |
| N3 | 1672.80 | 13717.87 | 9001.73 | 4088.26 | 0.45 |
| All | 703.84 | 15648.27 | 7734.67 | 4009.28 | 0.52 |

**TABLE 5.** Estimation results of rice AGB during the critical growth stages based on spectral and texture features derived from D2 direction and middle window size.

| **Stages** | **Metrics** | **Small window** | **Middle window** | **Large window** |
| --- | --- | --- | --- | --- |
| Late Tillering stage | RMSE(kg/ha) | 398.01 | 401.41 | 440.33 |
|  | R2 | 0.76 | 0.75 | 0.68 |
|  | rRMSE(%) | 21.34 | 21.52 | 23.60 |
| Booting stage | RMSE(kg/ha) | 1230.08 | 1316.81 | 1227.62 |
|  | R2 | 0.82 | 0.80 | 0.84 |
|  | rRMSE(%) | 14.28 | 15.29 | 14.26 |
| Heading to Flowering stage | RMSE(kg/ha) | 1746.90 | 1759.45 | 1842.97 |
|  | R2 | 0.57 | 0.59 | 0.54 |
|  | rRMSE(%) | 20.46 | 20.61 | 21.59 |
| Early Filling stage | RMSE(kg/ha) | 1944.12 | 2019.23 | 1969.79 |
|  | R2 | 0.56 | 0.51 | 0.54 |
|  | rRMSE(%) | 19.23 | 19.97 | 19.48 |
| All stage | RMSE(kg/ha) | 1803.83 | 1762.80 | 1750.56 |
|  | R2 | 0.82 | 0.82 | 0.83 |
|  | rRMSE(%) | 22.30 | 21.79 | 21.64 |


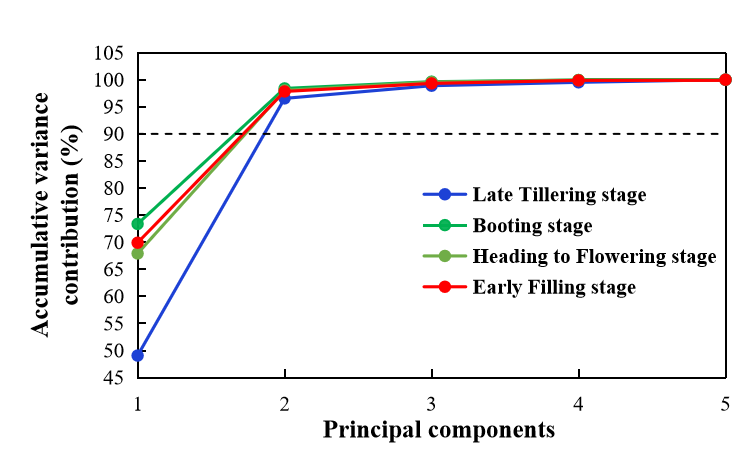


**FIGURE 1.** Accumulative contribution rate in principal component analysis of UAV multispectral images.
